# A meta-analysis of the validity of the Head-Toes-Knees-Shoulders task in predicting young children's academic performance

**DOI:** 10.3389/fpsyg.2023.1124235

**Published:** 2023-06-20

**Authors:** Sabrina Ann Kenny, Claire E. Cameron, Jasmine Tua Karing, Ahmad Ahmadi, Paige Noelle Braithwaite, Megan M. McClelland

**Affiliations:** ^1^Human Development and Family Sciences, Oregon State University, Corvallis, OR, United States; ^2^Department of Learning and Instruction, University at Buffalo, Buffalo, NY, United States

**Keywords:** self-regulation, executive function, meta-analysis, academic achievement, early childhood assessment

## Abstract

The present study represents the first meta-analytic synthesis of the utility of a widely used early-childhood self-regulation measure, the Head-Toes-Knees-Shoulders task, in predicting children's academic achievement. A systematic review of the literature yielded 69 studies accessed from peer reviewed journals representing 413 effect sizes and 19,917 children meeting the complete set of inclusion and exclusion criteria. Robust variance analysis demonstrated that the Head-Toes-Knees-Shoulders task was a consistent predictor of children's academic achievement across literacy, oral language, and mathematical outcomes. A moderator analysis indicated that in accordance with prior research, the Head-Toes-Knees-Shoulders task was more strongly associated with children's mathematics performance relative to their performance on language and literacy measures. The results of this meta-analysis suggest that the Head-Toes-Knees-Shoulders task demonstrated statistically significant, positive associations with children's overall academic performance. These associations remained stable across different participant and measurement factors and are comparable to meta-analyses examining the self-regulation and academic association with multiple measures of self-regulation and executive function.

## Introduction

Early childhood experts agree that children who can express their emotions and manage their behaviors to meet the demands of their classroom context begin school with an academic and developmental advantage (Korinek and deFur, 2016; McKown, 2017). Self-regulation, which is included under the self-management competency in the social and emotional learning literature (Collaborative for Academic, Social and Emotional Learning, n.d., para. 3), refers to the skills involved to exert control over one's own actions, thoughts, and feelings in accordance with the social expectations of the environment (Willoughby et al., 2012; Blair and Raver, 2015; Korinek and deFur, 2016; Nigg, 2017). Scholars suggest that the construct of self-regulation is multidimensional and involves both conscious (i.e., top-down) and unconscious (i.e., bottom-up) biopsychosocial processes (Best and Miller, 2010; McClelland and Cameron, 2012; Blair, 2016; Nigg, 2017). The conscious aspect of self-regulation depends greatly on three executive function (EF) components: working memory (the ability to hold and manipulate information in short-term memory), attentional flexibility (the ability to shift one's thoughts and attention when necessary), and inhibitory control (the ability to suppress a habitual response in favor of a more socially acceptable one; Best and Miller, 2010; Nigg, 2017; Gonzales et al., 2021). In the current study, we focus on these cognitive processes as they relate to children's behavioral responses in school, such as paying attention, ignoring distractions, and following directions while completing academic tasks. We are particularly concerned with the extent to which children can apply and integrate their executive functions into observable motor actions. However, we acknowledge that the conceptualization of self-regulation, executive functioning, and other related constructs may have slight variations across disciplines with different scholarly traditions and measurement practices (Booth et al., 2018).

Given the widespread consensus that self-regulation is essential to children's successful school participation (Rimm-Kaufman et al., 2009; McClelland et al., 2014; Blair and Raver, 2015, 2016; Edossa et al., 2018; Hernández et al., 2018; Robson et al., 2020; Finders et al., 2021), there has been a rise in self-regulation interventions across research and practice settings (Reid et al., 2005; Raver et al., 2011; Carr et al., 2014; Flook et al., 2015; Schmitt Et al., 2015; Pandey et al., 2018; McClelland et al., 2019; Hahn-Markowitz et al., 2020; Razza et al., 2020; Welsh et al., 2020). Nevertheless, the literature has continuously acknowledged the scarcity of high-quality, direct (i.e., performance-based) assessments of self-regulation suitable for young children and relevant to educational contexts (Raver et al., 2012; Halle and Darling-Churchill, 2016; Lipsey et al., 2017; McKown, 2017). A topic that has received much attention both in the U.S. and abroad has been on the validity of self-regulation measures in predicting outcomes deemed meaningful in school, most particularly, young children's academic achievement (e.g., Gestsdottir et al., 2014; McClelland et al., 2014, 2021; Schmitt et al., 2014, 2017; Lipsey et al., 2017; Hee et al., 2018).

One assessment tool used frequently in educational research is the Head-Toes-Knees-Shoulders task (HTKS; McClelland et al., 2007; Cameron Ponitz et al., 2008, 2009). The HTKS is a direct assessment of self-regulation that asks children to do the opposite of a series of gross motor actions (e.g., “touch your head when I say touch your toes”). If the child succeeds, more difficult instructions requiring rule-switching are introduced. A distinct advantage of the HTKS is that it emulates the real challenges of school, requiring children to regulate observable behavior by integrating all three aspects of EF simultaneously: (a) working memory because they listen to, remember, and respond to the multiple rules and commands; (b) inhibitory control because they must suppress their natural response to touch the body part they are being asked to touch; and (c) attentional flexibility because they focus on the multiple aspects of the task and transition between the old and new rules (McCabe et al., 2000, 2004; McClelland and Cameron, 2012; Gonzales et al., 2021). Thus, from a conceptual standpoint, the HTKS aligns well with contemporary, strength-based perspectives that emphasize the contextually and culturally situated nature of executive functioning (Doebel and Lillard, 2023).

The HTKS was developed in the U.S. and is now available in 28 languages. For over a decade, it has become a popular choice for early childhood researchers examining self-regulation in populations with diverse racial, cultural, and socioeconomic backgrounds (Wanless et al., 2011a; Gestsdottir et al., 2014; Lonigan et al., 2017; Zhang and Rao, 2017; Hee et al., 2018; Hernández et al., 2018). To date, the HTKS has been used primarily by researchers, but several of its characteristics make it a promising prospect for educational practice; these include a straightforward administration/scoring procedure, portability, no technology required, developmental appropriateness, and testing items that relate to what children are frequently expected to do in real-life school settings. The HTKS has been viewed as an ecologically valid self-regulation measure because of its focus on assessing classroom behaviors that help children navigate early learning contexts. In addition, the gross motor component of the HTKS more closely approximates children's behavioral self-regulation compared to other measures (McClelland and Cameron, 2012).

Several researchers have tested the reliability of the HTKS, and the data show strong psychometric properties with little variability across studies, including coefficient alpha, test–retest reliability, and interrater reliability (Cameron et al., 2012; McClelland et al., 2014; Lipsey et al., 2017; Zhang and Rao, 2017; Hee et al., 2018; Liu et al., 2018; Spiegel and Lonigan, 2018; Cerino et al., 2019; Sezgin et al., 2019). Some studies have also found the HTKS predictive of academic outcomes and significantly related to children's working memory, attentional flexibility, and inhibitory control (Cameron Ponitz et al., 2008, 2009; Gestsdottir et al., 2014; McClelland et al., 2014, 2021; Schmitt et al., 2014, 2017; Fuhs et al., 2015; Lipsey et al., 2017; Hee et al., 2018; Gonzales et al., 2021). Recently, the most current version of the HTKS, the HTKS-R, showed a significant increase in its ability to capture variability in children with lower levels of self-regulation compared to earlier HTKS versions (Gonzales et al., 2021).

Considering the frequent use of the HTKS in school-based research across various cultural contexts, it is critical to understand all the available evidence on the instrument's measurement characteristics, most particularly, its relevance to educational outcomes. Statistically synthesizing the results of several studies will provide researchers and practitioners with a cohesive representation of this knowledge. Because the HTKS has grown in use since the first empirical studies in 2007–09, it would be challenging to evaluate such a large body of literature objectively without a quantitative approach. Borenstein et al. (2009) warned that attempting to synthesize the results of numerous studies without meta-analytic techniques often leads researchers to make inferences that are not well-informed because such an approach relies so heavily on one's subjective reasoning and judgment. Similarly, without a systematic and transparent approach to database searching and study selection, the chances of overlooking relevant publications with the HTKS is high (Borenstein et al., 2009).

To provide a more objective estimate of the HTKS and academic association, the primary purpose of this study was to systematically gather and quantitatively combine all the available evidence on the association between children's (ages 3–8 years of age) HTKS performance and their academic achievement in mathematics, oral language, and literacy outcomes with meta-analytic techniques. Children's performance on mathematics, oral language, and literacy measures were the outcomes of focus in this study because these are the skills that are targeted most heavily in early childhood school settings. Other researchers have measured the behavioral regulation and academic association meta-analytically with multiple measures of self-regulation (Dent, 2013; Robson et al., 2020). These studies have provided valuable insights into self-regulation at the construct level, but are less valuable for making inferences related to self-regulation measurement. To our knowledge, this study represents the first attempt to examine the self-regulation and academic association meta-analytically with the HTKS only. Focusing on the measurement properties of the HTKS is significant because of how frequently researchers rely on the tool as an outcome measure.

### Variability in the HTKS and academic performance association

Meta-analysis is a quantitative design that uses statistical techniques to combine the results of existing studies related to a particular research topic or question (Borenstein et al., 2009; Wilson, 2019). In addition to providing a potentially more objective estimate of the association between children's performance on the HTKS and their academic achievement, a meta-analysis can uncover trends and patterns in the data that would otherwise be difficult to identify. For example, meta-analytic techniques can estimate the degree to which variability in effect size estimates stem from real effect size variation and identify factors that might be contributing to this variation (Borenstein et al., 2009). Unlike a traditional narrative review, a meta-analysis can, therefore, shed light on the circumstances and contexts where the HTKS performs best in predicting children's academic performance.

Variations in studies' findings can occur by chance, but several measurable factors can also potentially alter the magnitude or direction of the association between children's self-regulation and their academic performance. As mentioned earlier, researchers had meta-analyzed the association between children's academic performance and self-regulation on the construct level. The direction of the association remained positive and significant in these meta-analyses; however, some scholars have found the magnitude of the effect size to vary as a function of different participant and measurement factors (Dent, 2013; Robson et al., 2020). In the present study, we expected that the association between children's HTKS performance and their academic achievement would be positive and significant across studies but might vary as a function of the following participant, contextual, and measurement characteristics: average age of the sample, the country where the study took place, the academic subject domain, and the testing occasion. Understanding the degree to which the HTKS correlates with children's academic performance across different samples under various circumstances holds critical implications for educational researchers and practitioners seeking self-regulation measures that are consistently predictive of meaningful educational outcomes.

#### Variation by age

There is some evidence that age may moderate the association between children's self- regulation skills and academic performance. For example, in a meta-analysis spanning a wide age range of children and adolescents, Dent (2013) found the average association between children's self-regulation skills and their academic achievement to be significantly stronger in elementary school children than preschool children. Although the exact mechanisms by which children's age influences the association between their self-regulation skills and academic achievement are not fully understood, we hypothesized that age may moderate the HTKS and academic achievement association in the present study because the HTKS captures less variability in children with emergent self-regulation skills (Gonzales et al., 2021; McClelland et al., 2021). The HTKS was recently revised (i.e., HTKS-R) to address this concern, but the first publication of the HTKS-R measure was in 2019 (McClelland et al., 2019). Therefore, because most of the investigations to date of the HTKS relied on the original HTKS, we proposed that the strength of the HTKS and academic performance association might decrease as the average age of the samples decreases.

#### Variation by country

Several researchers outside the U.S. have found the HTKS predictive of children's academic performance (Lan et al., 2011; Wanless et al., 2011b; von Suchodoletz et al., 2013; Gestsdottir et al., 2014; Cadima et al., 2015; Birgisdottir et al., 2020; Lenes et al., 2020). Nevertheless, because society plays such a vital role in shaping how children behave in school, how teachers expect children to behave, and the academic demands of an early learning environment, it is plausible to suspect that the association between children's HTKS performance and their academic performance might vary from country to country (Wanless et al., 2011a,b; ten Braak et al., 2019). Cultural differences intersecting with measurement factors may also contribute to variations in the strength of the self-regulation and academic association. For example, scholars have suggested that children in China may perform better on self-regulation measures than their peers from other countries because of the Asian societal emphasis on self-discipline and behavioral control (Zhang and Rao, 2017). Thus, the ceiling effect on the HTKS may be more prominent in children from Asian countries; if the HTKS captures less variability in Asian children, it may yield weaker academic associations when used with this population.

#### Variation by academic domain

Cross-sectional and longitudinal research has shown that children with well-developed self-regulation, including its underlying executive functioning components, demonstrate more positive short and long-term outcomes in mathematics, literacy, and oral language (Dent, 2013; Fuhs et al., 2014; Cadima et al., 2015; Cantin et al., 2016; Lonigan et al., 2017; Purpura et al., 2017; Schmitt et al., 2017; Hernández et al., 2018; McClelland and Cameron, 2019; Valcan et al., 2020). The strongest relations have been found with mathematics (McClelland et al., 2014; Blair et al., 2015). One common explanation linking self-regulation to academic achievement relates to automaticity, a theory stating that through practice and repetition, most people will eventually become capable of performing specific skills with little-to-no conscious effort and thought (Wulf et al., 2001; Floyer-Lea and Matthews, 2005).

The concept of automaticity can shed light on why the strength of the HTKS and academic association might vary across different academic skills. In other words, academic skills can become automated and, therefore, necessitate less executive control (Cameron, 2018).

To illustrate, scholars proposed that although children will depend greatly upon all three key EF for early literacy (e.g., alphabet knowledge, decoding, and phonological awareness) acquisition initially, with practice and repetition, they will eventually be capable of applying these skills with little conscious effort given the relatively small amount of information to be acquired (Paris, 2005; Spiegel et al., 2021). Similarly, children often learn much of their vocabulary skills through social interactions as opposed to direct instruction (Fuhs et al., 2015). Given this frequent exposure, much like early literacy skills, oral language (e.g., vocabulary) acquisition may require less mental effort in early childhood than academic domains that continue to grow in complexity and novelty, such as mathematics (Blair et al., 2008; Fuhs et al., 2015). We, therefore, suspected that the association between children's HTKS performance and children's academic achievement would be strongest in mathematics.

#### Variation by testing occasion

Research on the self- regulation and academic achievement association includes cross-sectional studies and longitudinal studies with multiple waves of data collection. We hypothesized that cross-sectional studies might yield substantially stronger correlations between children's HTKS performance and their academic achievement due to common factors within testing situations that occur simultaneously or very close in time. In other words, test concurrency could inflate the association between children's HTKS performance and their academic performance.

### Research aims

The purpose of this study was to systematically gather a wide range of studies containing both HTKS and academic outcomes and to examine this data with meta-analytic methods. In so doing, we extended knowledge about one of the most frequently and globally used self-regulation direct assessments with the following research questions. Though these research questions were not pre-registered, we established them prior to beginning the meta-analysis.

1. What is the overall association between the HTKS and children's academic achievement and is there evidence of variability in this effect?
2. Do between-study differences in participant, contextual, and measurement characteristics influence the magnitude of the HTKS and academic achievement effect?
3. Do within-study differences in participant, contextual, and measurement characteristics influence the magnitude of the HTKS and academic achievement effect?

## Methods

The methods included in this meta-analysis, which are based on best practice guidelines (Borenstein et al., 2009; Kugley et al., 2017; Wilson, 2019; PRISMA, 2020), entailed (a) systematically searching for studies to address the pre-established research questions above; (b) screening the studies based on pre-established inclusion and exclusion criteria; (c) reviewing each study that met the criteria for the full text review to determine whether they met the full set of criteria for the quantitative analysis; (d) carefully reviewing and coding the studies to extract and understand the data; (e) calculating the individual studies' effect sizes and variances; (f) combining the studies to calculate a weighted mean (i.e., summary effect, average effect); (g) examining heterogeneity with a moderator analysis; (h) assessing study quality; and (i) testing for publication bias. These steps are presented in more detail below. To guide this meta-analysis, we followed the Preferred Reporting Items for Systematic Reviews and Meta-Analyses (PRISMA) to the greatest extent possible: “PRISMA is an evidence-based minimum set of items for reporting in systematic reviews and meta-analyses” (PRISMA, 2020, para. 1).

### Search process

Following the Campbell Collaboration's information retrieval guidelines (Kugley et al., 2017), in the winter of 2021 and the spring of 2022, the first author conducted several searches using ERIC, PsycINFO, Psychology and the Behavioral Sciences, and Web of Science. The search was restricted to the years 2007 through 2022 because HTKS publications did not exist before this time. To increase the chances of obtaining all relevant research with the HTKS instrument, the search terms included a Boolean, all-text search with the “or” operator combining all of the possible nomenclatures of the outcome measure (e.g., HTKS **OR** Head-Toes-Knees-Shoulders task **OR** Head Toes Knees Shoulders **OR** Head-to-Toes-Task). After this search, the first author cross-referenced the findings with a running list of HTKS studies; one of the authors of the HTKS, updates this list regularly to keep track of who is using the instrument. The first author also searched the publication reference lists of meta-analyses examining the association between children's self-regulation (and related constructs) and their academic performance.

### Study selection

To be included in the meta-analysis, the research had to meet the following full set of eligibility criteria: (a) provide a full text in English that is published in a peer-review journal; (b) report original data only (i.e., studies reporting coefficients from other studies such as meta-analyses or narrative reviews were excluded); (c) report quantitative data from the HTKS as an outcome measure; (d) report quantitative data from at least one math, literacy, or oral-language outcome; (e) provide one or more 0-order Pearson correlation coefficients between the HTKS and children's academic performance (either in the article or obtained from the researchers); (f) use a sample of children who were between 3 and 8 years old at the time of HTKS testing; (g) not use an unconventional HTKS administration approach, such as a gamified version; and (h) not exclusively target children with developmental delays, disabilities, or emotional and behavioral challenges. For academic outcome measures, both standardized assessments and teacher reports or ratings were considered acceptable. Regarding design, both cross-sectional and longitudinal studies were included. In addition to non-experimental/observational studies, studies examining the effects of interventions were included when the correlations at baseline (prior to intervention) or for the control group were available in the article or from the researchers.

The final comprehensive search process yielded 403 studies (*k* = 218 from PsycINFO, *k* = 70 from Psychology and the Behavioral Sciences, *k* = 76 from Web of Science, and *k* = 39 from ERIC). After the first author removed all duplicates (e.g., same studies appearing across multiple databases), *k* = 311 studies remained. This total included an additional 19 studies that were not found via the database search; the first author accessed the 19 publications via the previously mentioned running list of HTKS studies and the reference lists of relevant meta-analyses. Using a pre-established abstract screening checklist (available upon request from the corresponding author), the first author screened all records independently; a random sample of ~15% of the studies were then selected so that a team of two screeners (trained by the first author but blinded to her screening decisions) could conduct a double screening using the same abstract screening protocol. All three screeners reached an agreement level of 98 percent over which studies qualified for the full-text review.

The abstract screening process resulted in the elimination of 120 studies for one or more of the following reasons per the abstract screening checklist: the article contained no HTKS outcome; the article contained no academic outcome; the sample included adults or adolescents only; the article was a literature review or meta-analysis; the full paper was not available in English; the paper was not published in a peer reviewed journal. Following the abstract screening, the first author read each of the remaining articles (*k* = 191) with a pre-established, full-text eligibility checklist stating the eligibility criteria for the quantitative analysis (available upon request from the corresponding author). Like the abstract screening process, a team of two assessors trained by the first author but blinded to the first author's eligibility decisions assessed a random sample of 15% of these remaining articles using the same full-text eligibility protocol. All three screeners reached an agreement level of 93% about which studies qualified for the meta-analysis. A written record of disagreements and resolutions is available upon request from the corresponding author.

After the full-text review process, including attempts to obtain relevant missing data from the corresponding authors of studies with no 0-order correlations, the next step was to ensure that each study's correlations were based on a distinct sample. Therefore, the first, fourth, and last author conducted a sample comparison across all studies to assure the independence of each sample. Prior to analysis, the first author removed all duplicate datasets and studies in which the correlations could not be obtained.

**Figure 1:**
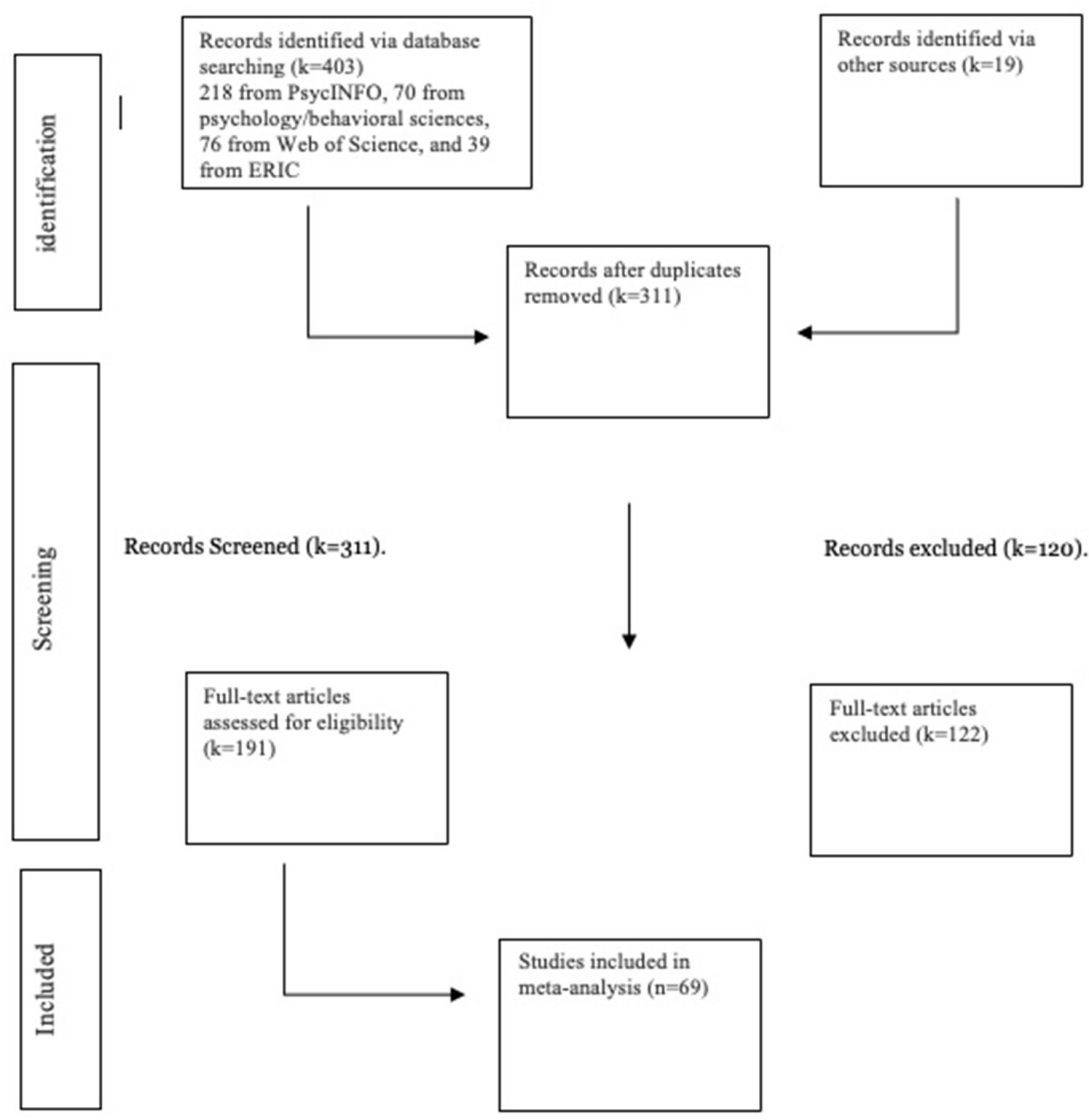
Flow chart of study selection process.

The full inclusion/exclusion process resulted in a total of *k* = 69 studies eligible for the quantitative analysis. A reference list of the included studies is in Appendix A in the Supplementary material. The reference list of excluded studies with the reasons for exclusion is in Appendix B in the Supplementary material. A PRISMA flow diagram outlining the different phases of the review is presented in Figure 1. In both the abstract screening and full-text inclusion/exclusion process, all assessors resolved 100% of their discrepancies via a discussion and review of the eligibility criteria.

### Data extraction

In addition to extracting each study's identifying information (e.g., authors, title, publication year, and journal) and the information needed to calculate the effect size (analytic sample size and 0-order correlations), the first author coded several study-level characteristics. Additionally, each study was given a score representing its overall study quality. All coding was conducted via a formal coding protocol developed by the first author (available upon request). All moderators and effect size coding were entered into Excel and checked for data entry errors prior to transferring it into Stata for the primary analysis. The first author also checked for data entry errors in Stata by comparing descriptive statistics calculated in both Stata and Excel.

#### Moderator coding

In a meta-analysis, a moderator analysis uses statistical techniques like ordinary least squares regression (i.e., meta-regression) in the attempts to identify study-level factors that contribute to differences in the outcome of interest (i.e., the association between children's HTKS performance and their academic achievement). We selected the variables described below as effect-size moderators because (a) they pertained to the pre-established research questions; (b) were reported consistently and completely throughout the papers, and (c) demonstrated noticeable variation across papers.

##### Age

We initially recorded the mean age of the participants in whatever way the researchers reported it (e.g., months or years). For the final analysis in Stata, age was represented by the average age of the sample in months at the time of HTKS testing. Per our inclusion/exclusion criteria, we only included studies and timepoints where the average age of the sample was between 36 and 96 months old at the time of HTKS data collection.

##### Country

During the initial coding process, we recorded the country where each study took place. However, for the final analysis, the country variable was collapsed into the following four categories: Asian countries, European countries, United States, and a category signifying countries outside of Asia, Europe, and the United States (i.e., other).

##### Testing occasion

Because of our choice to include both cross-sectional and longitudinal data, an important measurement characteristic of the HTKS was the time it was administered to children relative to the academic achievement testing. This meta-analysis contains effect sizes representing the HTKS and academic performance association where (a) both the HTKS and academic testing occurred at the same time point or where (b) the HTKS was administered to children prior to the academic testing (e.g., HTKS administered in the fall of preschool followed by kindergarten academic achievement testing). To capture this variation, initially, we recorded all the time points when the HTKS was administered and all the time points when the academic measures were administered. However, due to a low percentage of responses representing each of these categories, this variable was treated dichotomously for the moderator analysis representing either: (a) the HTKS administered at the same data collection wave as the academic testing and (b) the HTKS administered prior to academic testing (i.e., HTKS administered at time 1; academic measures administered at time 2).

##### Academic domain

Informed by prior literature and how studies reported the correlations between the HTKS and the academic achievement measures, we initially created an academic domain variable consisting of the following categories: literacy skills (e.g., phonological awareness, print awareness, letter and word identification, letter-sound knowledge, and decoding); oral-language skills (e.g., expressive and receptive vocabulary, listening comprehension, grammar, and syntax); and mathematics (all skills related to numbers/cardinality, operations, measurement, geometry, and quantitative problem solving). These categories aligned with most of the academic achievement measures in this meta-analysis and other common taxonomies for classifying literacy, oral-language, and mathematics skills (National Governors Association Center for Best Practices Council of Chief State School Officers, 2010a,b; U.S. Department of Health, 2015; Lonigan and Milburn, 2017). The only exception was when researchers reported children's academic performance globally (e.g., a single academic score representing math, literacy, and oral-language). This type of outcome could not be classified into one of the subject-specific domain categories. Therefore, we classified such measures as academic global (i.e., math/language/literacy composite) and language arts global (i.e., language and literacy composite).

For the moderator analysis in Stata, the academic domain variable was transformed into a dichotomous variable representing content that was either math related or language arts related because there were not enough outcomes in each of the initial categories above. For the dichotomous variable, the term language arts refers to all literacy, oral language and language arts global measures collectively. For construct clarity purposes, the academic global measures were dropped from the moderator analysis, resulting in a marginal sample size reduction.

Using a detailed coding protocol, a team of two coders (trained by the first author but blinded to her coding decisions), double coded a random sample of ~15% of the studies and effect sizes representing each of the moderators above. Level of agreement among all three coders was 100% for each variable across all studies.

##### Study quality assessment

After reviewing several rating scales and study quality indicators, we used a scale informed by the one that Zangaro and Soeken (2005) developed in their meta-analysis of the reliability and validity of a job satisfaction questionnaire. We revised this scale to include 10 items reflecting what was most critical for examining the validity of the HTKS in predicting children's academic achievement, namely how well the researchers described the academic and HTKS testing and scoring procedure (available in Supplementary material). The range of total quality points was 0–10.

To code for study quality, the first and third author divided the studies and rated each study using the previously mentioned quality rating scale. Following this process, the first and third author double-coded a random selection of ~15% of each other's studies. The average level of agreement across all 10 items was 93%. However, both authors were able to resolve 100% of these disagreements via a discussion and review of a quality rating scale codebook developed by the first author. All sources of disagreement and their resolutions are available upon request.

#### Effect size coding

For this meta-analysis, the Fisher *Z*-transformation of the correlation coefficient served as the effect size variable. Each study has at least one effect size representing the association between children's HTKS performance and their academic achievement. To calculate the effect size, we extracted the 0-order Pearson's correlations and analytic sample sizes from the studies. The first author then used this information to transform each correlation to Fisher's *z* and calculated the variance for each of these estimates with an effect size calculator offered by the Campbell Collaboration. Using a detailed coding protocol (which included instructions for extracting the sample size, 0-order correlations, and converting the 0-order correlations to Fisher *Z*-transformation), the same coding team double-coded a random sample of ~15% of studies representing all effect size variables: sample size, Pearson's correlation, Fisher *Z*-transformation, and variance. Apart from sample size, level of agreement was 100% among the three coders. For sample size, level of agreement was 96% due to one coder's overlook regarding the analytic sample of the study.

### Data synthesis and statistical analysis

In the social and behavioral sciences, it is common for a single study to report multiple outcomes that may be of interest to the meta-analyst; yet, traditionally, most meta-analysis techniques have required each effect size to correspond to only one distinct sample. This guideline, although limiting, safeguards against the issue of assigning more weight to a study just because it has more effect sizes (Borenstein et al., 2009).

Meta-analyst scholars have recently determined that robust variance estimation (RVE) is one of few methodologically acceptable ways to combine multiple, statistically dependent outcomes, even when both the source and extent of the dependencies are not fully known (Hedges et al., 2010; Tanner-Smith and Tipton, 2013; Moeyaert et al., 2017; Cheung, 2019; Pustejovsky and Tipton, 2021). In addition to synthesizing more than one effect size in a single study, researchers using RVE can statistically separate and examine both between-study and within-study moderator effects. For example, most commonly, the average age of the sample will vary from study to study (i.e., between-studies) because each study represents different samples of children. However, the average age of the sample can also vary within the studies themselves (e.g., children's age will be different at fall and spring data collection); these two types of variation (i.e., between studies and within studies) could obscure one another's effects in a moderator analysis if they are not accounted for (Tanner-Smith and Tipton, 2013).

In this study, most effect-size dependencies stemmed from researchers collecting data over different time points with the same group of children, researchers reporting more than one academic outcome on the same group of participants, or a combination of these two types of dependencies. We used Robumeta packages offered by Stata 17 (small sample correction feature turned on) with a probability level set at 0.05, random model weights, and a correlated effects model. Robumeta has been endorsed by the Campbell Collaboration (Tanner-Smith and Tipton, 2013; Pustejovsky and Tipton, 2021). Researchers have found the technique to be robust to normality violations and effective for handling both small and large sample sizes (Pustejovsky and Tipton, 2021).

After data cleaning, descriptive analysis, and outlier testing, the first author calculated the weighted summary effect for all the outcomes combined by estimating an intercept-only model. This analysis was followed by two additional intercept-only models that combined effect sizes representing math measures only and effect sizes representing language arts measures only. The tau-square statistic was used to examine heterogeneity among studies' effect sizes, and Edger's test was used to assess potential publication bias. Because the tau-square statistic suggested some heterogeneity, these analyses were followed with statistical significance testing (three multivariable meta-regression models with a *p*-value of 0.05) to examine whether the HTKS and academic achievement association varied as a function of the previously discussed moderators.

## Results

First, we discuss the characteristics of the 69 studies of this meta-analysis. Following this descriptive analysis (Table 1), we present the meta-analytic findings related to the three research questions (Tables 2, 3).

**Table 1:** Descriptive analysis.

**Variables**	***n***	***k***	***M or %***	**SD**	**Range**
**Country**					
US	237	32	57%		
Asia	56	9	14%		
Europe	73	15	18%		
Other	47	14	11%		
Male	381	65	50.06%	3.77%	38.33–63%
Age	413	69	63.22	11.25	37–95.87
Study quality	413	69	8.75	1.33	6–10
**Academic domain**					
Math	138	44	33.41%		
Literacy	145	44	35.11%		
Oral language	107	42	25.91%		
Language arts global	16	6	3.87%		
Academic global	7	6	1.69%		
**HTKS testing occasion**					
Same time as academic assessment	258	66	62.47%		
Before academic assessment	155	33	37.53%		
Sample size	413	69	415.69	465.34	17–2,406

Total number of effect sizes: (n = 413). Total number of studies: (k = 69).

Means (M), percentages (%), standard deviations (SD), and ranges are based on the total number of effect sizes extracted from studies used in this meta-analysis.

**Table 2:** Average weighted effects for the association between the HTKS and children's academic performance.

**Variables**	***k***	***N***	***z***	**95% CI (LL)**	**95% CI (UL)**	***R***	***p-*value**	**Tau-squared**
All measures combined	69	413	0.4	0.37	0.44	0.38	< 0.001	0.0197
By academic domain								
Math	44	138	0.49	0.45	0.53	0.46	< 0.001	0.0143
Language arts	61	268	0.35	0.33	0.38	0.34	< 0.001	0.0122

k, number of studies; n, number of effect sizes; CI, confidence interval; LL, lower limit; UL, upper limit; z, Fisher z.

Language arts = all oral language, language arts global, and literacy measures combined into a single category.

**Table 3:** Moderator analysis.

**Variables**	**Model 1**	**Model 2**	**Model 3**
Study quality	−0.0026 (0.0112)		
Age between		−0.0001 (0.0019)	
Academic domain between (math = ref coded as 0)		^**^−0.2089 (0.0722)	
**Country (US** = **ref category coded as 0)**			
Asia		−0.0700 (0.0434)	
Europe		−0.0149 (0.0442)	
Other		−0.0555 (0.0505)	
HTKS testing occasion between (same time as academics = ref coded as 0)		−0.0432 (0.0544)	
Age within			0.0039 (0.0026)
Academic domain within (math = ref coded as 0)			^***^−0.1288 (0.0139)
HTKS testing occasion within			-0.0272 (0.0135)
Number of effect sizes	413	406	406
Number of studies	69	63	63

Standard error in parenthesis.

^***^p < 0.001, ^**^p < 0.01.

### General description of the papers included in the meta-analysis

We extracted 413 effect sizes from 69 studies representing a total of 19,917 children. These studies were all published in peer reviewed journals; thus, the following results may change if gray literature had been included in the analysis (e.g., dissertations, conference papers).

The number of effect sizes per study ranged between one and 30 with six effect sizes (on average) reported per study. Most papers (over 80%) reported more than one effect size representing the HTKS and academic performance association over different periods of data collection, the HTKS and academic performance association over different academic domains, the HTKS and academic performance association representing more than one distinct group of children, or a combination of these three types of dependencies.

#### Sample characteristics

There was a wide range of sample sizes reported (17–2,406) with an average sample size of ~416 across all effect sizes. The cultural contexts of the samples were diverse: 237 effect sizes (57%) from 32 papers originated from children in the United States; 176 effect sizes (43%) from 38 papers came from children living in countries outside of the United States, most commonly European and Eastern Asian countries. Across effect sizes, the average age of the children was 63.22 months (*SD* = 11.25) or a little over 5 years old at the time of HTKS testing. Most of the effect sizes (~95%) were extracted from studies where the average age of the sample was at least 48 months (4 years old) at the time of HTKS testing, though children's age ranged from 37 to 95.87 months (~3–8 years old). The gender composition was split relatively equally between males and females in most studies.

#### Measurement characteristics

Both the HTKS and academic measures were typically administered by the researchers in a quiet area of the school setting. Some researchers administered multiple measures of self-regulation, whereas others administered the HTKS only. Academic measures most frequently consisted of well-known, standardized assessments of achievement (e.g., Woodcock-Johnson-III Academic Achievement Test, Peabody Picture Vocabulary Test, Test of Preschool Early Literacy, and Bracken School Readiness Assessment) and modified versions of these measures. Three studies used teacher ratings of children's academic performance instead of direct assessments. These were combined with the directly assessed academic performance measures given the moderate to high associations between proxy ratings and direct measures of academic achievement (Hoge and Coladarci, 1989; Feinberg and Shapiro, 2003; Südkamp et al., 2012; Mack et al., 2023). The HTKS was administered at the same time as the academic measures across 62% of the effect sizes, whereas 38% of the effect sizes represented HTKS and academic performance associations where the HTKS was administered before academic measures. As evident in Table 1, most effect sizes (over 90%) represented academic outcome measures across either the math, literacy, or oral language domain, while a small portion of effect sizes represented children's academic performance more globally.

#### Study quality

Study quality was rated on a scale of 0–10 (lowest quality to highest quality) with items deemed relevant to the focus of this meta-analysis. The papers in this meta-analysis had an overall score ranging from 6 to 10 (*M* = 8.75, *SD* = 1.33).

### HTKS and academic performance association: meta-analytic findings

Each of the effect sizes that we extracted for this meta-analysis represented the continuous association (Pearson's *r* converted to Fisher's *z*) between children's HTKS performance and their academic achievement on an assessment of one of five possible content areas: mathematics (*n* = 138), literacy (*n* = 145), oral-language skills (*n* = 107), language arts global (*n* = 16), and academic global (*n* = 7).

Borenstein et al. (2009) explains that combining different but conceptually related outcomes in meta-analysis (e.g., outcomes that represent different academic subjects, outcomes that provide information about different social and emotional domains) is permissible, as long as the findings relate to the research questions. Because we were interested in the association between the HTKS and children's overall academic performance, we ran one intercept-only meta-regression model to examine the weighted summary effect for all the outcomes combined (overall effect synthesizing all 413 effect sizes). However, because we were also interested in the domain-specific effects of the HTKS on children's academic performance, we ran two additional intercept-only models that meta-analyzed effect sizes separately according to whether the academic outcome was math related (*n* = 138) or language arts related (*n* = 268). The findings are displayed in Table 2 and discussed under research question (RQ) 1.

To examine whether the HTKS and academic performance association varied as a function of the different effect size characteristics, we performed statistical significance testing with three multivariate meta-analytic regression models. These findings are displayed in Table 3 and discussed under RQ 2 and RQ 3.

#### Relations between the HTKS and academic performance

The first research question focused on the overall association between the HTKS and children's academic performance, and evidence of variability in this association. Table 2 presents the weighted summary effect (Fisher *z*) and 95% confidence intervals for the association between the HTKS and children's academic performance across all academic measures and for the two additional intercept-only models (i.e., math only and language arts only). The weighted summary effect when effect sizes representing all measures were included (*n* = 413, *k* = 69) in the model was *z* = 0.4 with a 95% confidence interval ranging between 0.37 and 0.44. Because the 95% confidence interval is relatively narrow for this effect and does not include 0, these findings can be interpreted as a medium summary effect that is positive, precise, and highly significant (*p* < 0.001). As evident in Table 2, the average effect for the math only measures (*n* = 138, *k* = 44) was slightly higher (*z* = 0.49). For the language arts only measures (*n* = 268, *k* = 61), the average effect was slightly lower (*z* = 0.35) than the summary effect for both the math only measures and when effect sizes representing all measures were combined. Nevertheless, each of these separate analyses maintained the significance level (*p* < 0.001) despite this sample size reduction. Although it is impossible to eliminate all publication bias in a meta-analysis, Edger's test showed no statistically significant small study effects among this particular pool of studies. As illustrated in Table 2, the tau-square value for the summary effects of all three analyses suggests the effect size variability in this meta-analysis stems from true variability (i.e., more than just error).

**Figure 2:**
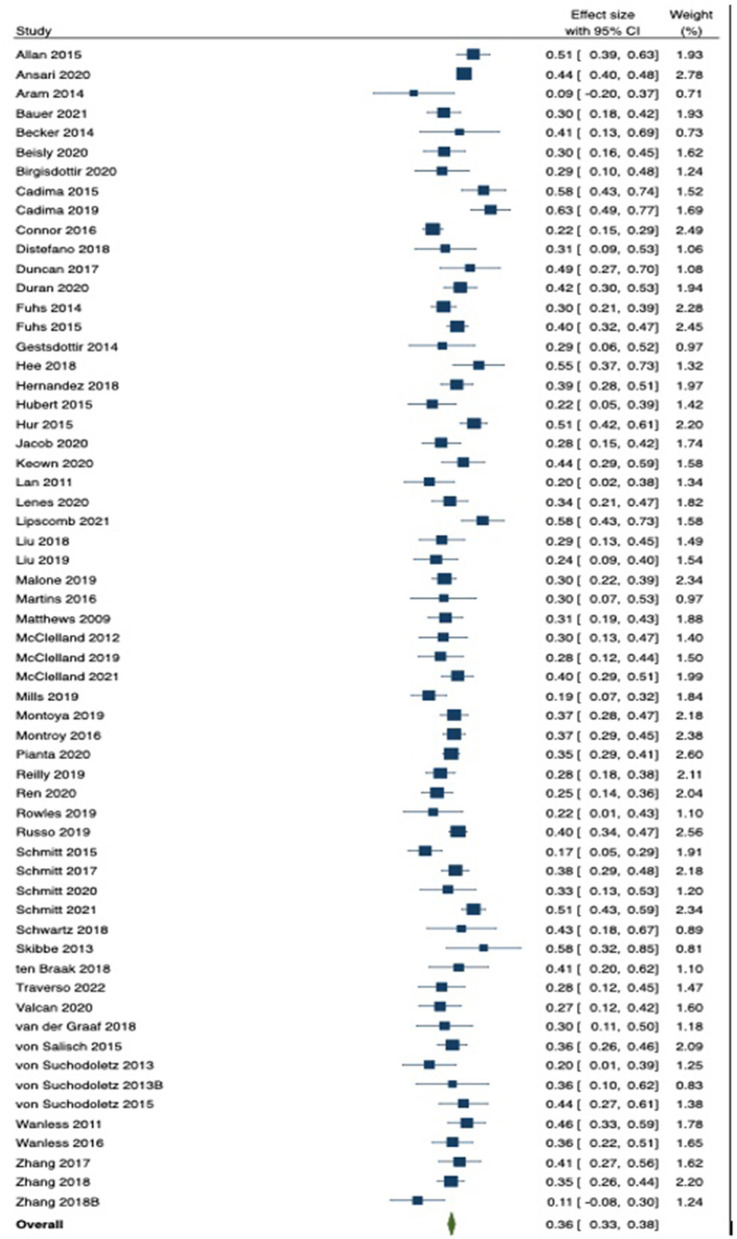
Forest plot for the HTKS and language arts association.

**Figure 3:**
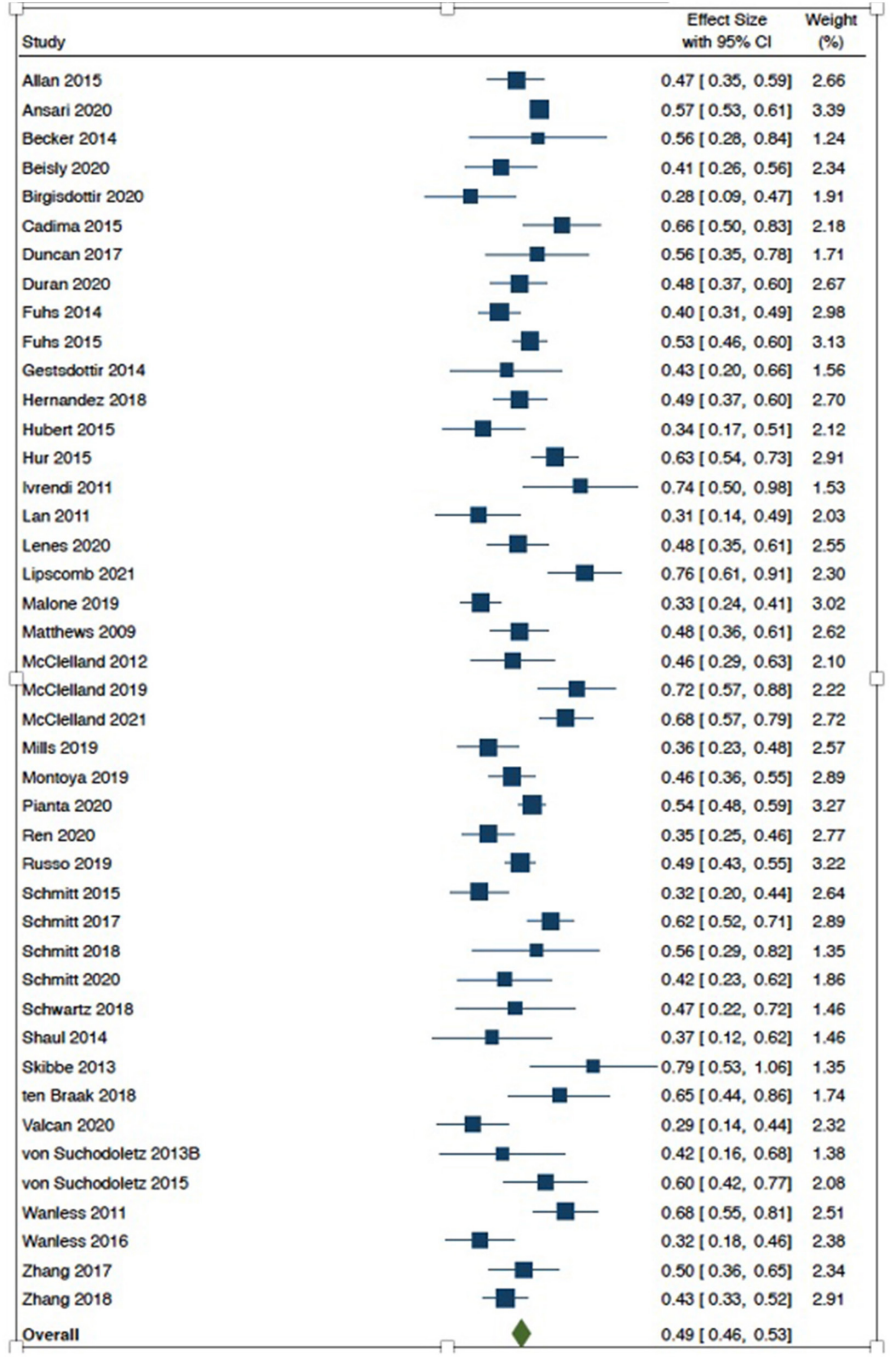
Forest plot for the HTKS and mathematics association.

The forest plots in Figures 2, 3 provide an illustration of how each study's effect size relates to the overall summary effect. Figure 2 includes studies with effect sizes representing the HTKS and language arts association, whereas Figure 3 includes studies with effect sizes representing the HTKS and mathematics association. As illustrated in the plots, on average, effect sizes representing the HTKS and mathematics association were noticeably larger than effect sizes representing the HTKS and language arts association. For readability purposes, studies with multiple effect sizes are represented by the mean of those effect sizes in the forest plots. A forest plot with all 413 effect sizes (robumeta plot) is available in the Supplementary material.

#### Sources of variability between and within studies

Research questions 2 and 3 focused on examining sources of variability in the effects between and within studies. Traditionally, the goal of a moderator analysis is to try to provide explanations for variations in effect sizes across the studies of a meta-analysis (Borenstein et al., 2009). However, effect sizes can vary both between and within the studies themselves. Thus, RQ 2 focused on examining between-study variation whereas RQ 3 focused on examining within-study variation. In all of the following meta-regression models, the effect size representing the HTKS and academic performance association serves as the outcome variable, and the moderators serve as the predictor variables. We used study means to estimate between-study effects of moderators (RQ 2) and study mean-centered values of moderators to estimate the within-study effects (RQ 3). Prior to running these models, we regressed the effect size variable on study quality to ensure the quality of a study would not influence the results. Based on the non-significant *p*-value of 0.82 and small regression coefficient of −0.0026, we concluded that study quality was an unlikely moderating factor between studies. Thus, it was not included in the subsequent multivariate regression models.

##### Variability in effects between studies

Research question 2 focused on whether between-study differences in participant and measurement characteristics influence the magnitude of the HTKS and academic performance effect. To explore heterogeneity in effects by between-study participant and measurement characteristics, we regressed the effect size variable (each study's Fisher *z*) on country (US = Ref coded as 0), age (continuous variable represented by the average age of the participants in months), academic domain (math = Ref coded as 0) and testing occasion (same as academic testing = Ref coded as 0). As evident in Table 3, when controlling for children's age, country, and testing occasion, on average, academic measures that were math related had statistically significantly stronger effect sizes than those that were language arts related between studies (*p* = 0.0078, *b* = −0.21). All the other variables in the model yielded a *p*-value > 0.05.

##### Variability in effects within studies

Research question 3 examined whether within-study differences in participant and measurement characteristics influence the magnitude of the HTKS and academic performance effect. To explore heterogeneity in effects by within-study participant and measurement characteristics, we regressed the effect size variable (each study's Fisher *z*) on age (continuous variable represented by the average age of the participants in months), academic domain (math = Ref coded as 0), and testing occasion (same as academic testing = Ref coded as 0). Like RQ 2, when controlling for children's age and testing occasion, on average, academic measures that were math-related had significantly stronger effect sizes than those that were language arts related when compared within studies (*p* < 0.001, *b* = −0.13); the other variables in the model yielded a *p*-value > 0.05.

## Discussion

This study found evidence that the HTKS predicts academic outcomes targeted in early learning contexts. When we analyzed the correlations between children's HTKS performance and their academic achievement across 413 effect sizes from 69 studies, the HTKS demonstrated significant and relatively consistent positive associations with children's current and subsequent performance across math, literacy, and oral language outcomes. After conducting the primary meta-analysis with all the measures combined (413 effect sizes), we performed two separate exploratory meta-analyses across the math related and language arts related measures. Like the meta-analysis with all the academic measures combined, the summary effects of these different subject domains, although strongest in math, remained positive and statistically significant when they were meta-analyzed separately. Taken together, these findings confirm prior research suggesting that self-regulation, including the previously mentioned EF components, make both domain-general and domain-specific contributions to children's academic performance, with associations generally strongest for mathematics (Dent, 2013; Allan et al., 2014; Fuhs et al., 2014; McClelland et al., 2014; Schmitt et al., 2017; Cortés Pascual et al., 2019; Birgisdottir et al., 2020; Robson et al., 2020).

Our findings per the moderator analysis demonstrated that except for the subject domain variable, the HTKS and academic association was relatively stable across different participant, contextual, and measurement factors. In other words, although on average, the HTKS was most predictive of children's math performance, no statistically significant effect size differences emerged across children's age, country, and whether the HTKS was given before or concurrent with achievement measures. Considering the widespread international use of the HTKS in research, along with the potential it holds for educational practice, this study makes a valuable contribution to the existing self-regulation literature. We propose that the findings have two critical implications: (a) they provide additional insights into some of the predictive properties of a widely-used self-regulation measure and (b) they reinforce existing research demonstrating the importance of self-regulation for early academic achievement.

### Predictive properties of the HTKS

A chief concern in the measurement of self-regulation is the need to identify instruments feasible for school settings that predict outcomes deemed meaningful in school (Lipsey et al., 2017; McCoy, 2019). One outcome deemed meaningful in school is children's academic performance. The results of this meta-analysis found the HTKS to demonstrate associations with children's academic performance that were comparable to and in some cases, slightly stronger than, the associations determined by meta-analyses using multiple measures of self-regulation and related constructs (Dent, 2013; Allan et al., 2014; Jacob and Parkinson, 2015; Cortés Pascual et al., 2019; Robson et al., 2020; Spiegel et al., 2021). This finding offers a valuable extension of Lipsey et al.'s (2017) study that identified the HTKS as one of the most robust performance-based, self-regulation measures for predicting children's academic achievement across the preschool and kindergarten years. One factor distinguishing the present study from Lipsey et al.'s work is that given the present study's meta-analytic methods, we were able to shed light on some of the conditions in which the HTKS performs best regarding its ability to predict children's academic performance across and within the studies via a moderator analysis. This knowledge is critical for researchers and practitioners seeking assessments with predictive properties that are reliable and valid in various contexts and populations.

The present study's moderator analysis suggested that the HTKS and academic association was relatively stable across different participant and measurement factors. In other words, although on average, the HTKS was more strongly associated with children's math performance, effect sizes remained positive and significant across the language and literacy domain as well. Furthermore, no statistically significant effect size differences emerged across children's age, country, and whether the HTKS was given before or concurrent with achievement measures. Below, we propose explanations related to the results of the moderator analysis with a focus on each of those four variables.

#### Country

One characteristic distinguishing this meta-analysis from meta-analyses examining the self-regulation and academic performance effect across multiple measures is that we examined how differences in participants' country impacted the association. The fact that the HTKS is available in 28 languages and has been used cross-culturally made this possible. The present study did not find the HTKS and academic performance effect to be moderated by the different countries represented. This finding is understandable given that researchers have consistently found evidence for both the stability and variability of the association between children's HTKS performance and their academic achievement across cultures (Lan et al., 2011; Wanless et al., 2011b; von Suchodoletz et al., 2013; Gestsdottir et al., 2014). The present study extends that research by demonstrating that when these studies are synthesized and analyzed meta-analytically, there is more evidence for the stability in children's need for self-regulation to succeed academically cross-culturally. One explanation for our findings is that the HTKS does not depend on teachers' appraisal of children's behaviors, which may explain why it appeared to capture the self-regulation and academic association relatively the same, regardless of the children's country. The HTKS may have yielded similar associations with children's academic performance across countries because it may be tapping into an aspect of the construct that is less context-or reporter-specific and more dependent on child-level factors.

#### Age

Although some research has found that age moderates the association between children's self- regulation and academic performance (Dent, 2013), our findings suggest that the HTKS predicts children's academic performance, regardless of the sample's average age. However, it is important to note that this lack of statistical significance may be due to a lack of variability in age, rather than the absence of a moderating effect. For example, despite the wide age range represented (ages 3 through 8) in our inclusion criteria, most of the effect sizes (~95%) were extracted from studies where the average age of the sample was at least 48 months (4 years old) at the time of HTKS testing. Because some researchers have found the HTKS to capture less variability in children with nascent self-regulation skills (Gonzales et al., 2021; McClelland et al., 2021), it is plausible to suspect that the HTKS and academic association may have been weaker if we had more samples representative of younger children (e.g., average age younger than 4 years old).

#### Testing occasion

While many researchers have found children's self-regulation to be associated with their academic performance, aspects of this relationship remain unclear even when controlling for various factors. One remaining question relates to whether the association is truly predictive, and if so, what assessments are most suitable for predicting children's academic performance (Lipsey et al., 2017). As recently mentioned, in a longitudinal study, Lipsey et al. (2017) ranked the HTKS as one of the most robust self-regulation measures for predicting children's academic performance across the preschool and kindergarten years. The fact that the present study found cross-sectional effect sizes, on average, not to statistically significantly differ from effect sizes in which the HTKS was administered prior to academic testing provides some corroborating support for Lipsey et al.'s conclusion. However, future research should investigate how unexplored factors may contribute to the stability of this effect. One possibility is that the time-lapse between the HTKS and academic data collection in the present meta-analysis was too narrow to represent effect size variability between cross-sectional and multi-waved studies. For example, most studies with multiple waves included fall and spring testing only. Thus, the results of this meta-analysis alone do not provide strong enough evidence to draw firm conclusions about whether the strength of HTKS and academic association varies by testing occasion, nor whether the association is truly predictive.

#### Academic domain

The present study's results indicated that, on average, the association between children's HTKS performance and their mathematics achievement was statistically significantly stronger than the association between children's HTKS performance and their language arts achievement. Although the exact mechanisms behind this relationship are unclear, some researchers have proposed that the self-regulation and academic achievement association is strongest in mathematics because mathematical tasks are the least familiar and more difficult to automate for most children (Blair et al., 2008; Fuhs et al., 2014; McClelland and Cameron, 2019; Spiegel et al., 2021). However, other plausible explanations should be considered. For example, it is possible that the mathematics measures in this meta-analysis had more sensitivity than the language and literacy measures. We are unable to provide a firm explanation as to why the HTKS and academic association was strongest for mathematics, as investigating the mechanisms behind this relationship was beyond our study's scope. Nevertheless, the finding that the self-regulation and academic achievement association was strongest in mathematics is consistent with prior research (Dent, 2013; Allan et al., 2014; Fuhs et al., 2014; McClelland et al., 2014; Schmitt et al., 2017; Cortés Pascual et al., 2019; Birgisdottir et al., 2020; Robson et al., 2020).

### The importance of self-regulation for early academic achievement

In addition to providing deeper insights into the predictive utility of the HTKS, this meta-analysis also sheds light on the self-regulation and academic association at the construct level. To our knowledge, no researchers have performed a meta-analysis focused on examining the self-regulation and academic association with a single measure. However, several researchers have synthesized this relationship on the construct level; these meta-analyses included multiple measures of self-regulation and related constructs, such as EF (Dent, 2013; Allan et al., 2014; Jacob and Parkinson, 2015; Cortés Pascual et al., 2019; Robson et al., 2020; Spiegel et al., 2021). The results of this meta-analysis found the HTKS to demonstrate associations with children's academic performance that were comparable to and in some cases, slightly stronger than these prior meta-analyses. Regardless of the particular measures, researchers are consistently finding evidence that self-regulation is a critical skill that children need for successful school participation. The significant, positive self-regulation and academic performance associations found across all the subject domains in the present study (coupled with the findings across these prior meta-analyses) confirm social and emotional learning advocacy efforts encouraging schools and classrooms to make teaching self-regulation to children a priority. In other words, if self-regulation is a consistent predictor of children's academic performance, to maximize all students' learning, schools may need to consider going beyond just teaching academics.

There is theoretical and empirical evidence showing that children's self-regulation and EF skills can improve over time in response to positive environmental supports and strategies (Blair, 2002; McClelland et al., 2010, 2015; Moffitt et al., 2011; Blair and Raver, 2015; Howard and Williams, 2018). Given the malleability of self-regulation (Blair and Raver, 2012; Morrison and Grammer, 2016) and the stability of the associations found in the present study, early childhood educators and practitioners should consider focusing more on promoting children's self-regulation and their related EF skills as one way to support their social-emotional growth and academic learning. This need has become increasingly pressing with school shutdowns and the stress and trauma children and families have been experiencing during the COVID-19 pandemic. A self-regulation assessment that is predictive of academic outcomes across differing cultural contexts can help researchers and practitioners monitor children's response to such interventions and help to inform school-based practices that can facilitate optimal school participation for diverse populations of children.

## Limitations and directions for future researchers

Several limitations bear mentioning. First, while 69 studies are considered sufficient for RVE, a larger sample would have allowed for a more expansive moderator analysis. Because we were limited to the number of moderators, we had to prioritize which variables made the most sense to examine, both methodologically and theoretically. For example, we decided not to include gender as a moderator because, during data cleaning, the descriptive analysis showed that the proportion of males to females across studies showed little variation across samples. In other words, the gender composition was split relatively equally between males and females in most studies. Like gender, we were unable to examine race/ethnicity or socioeconomic status because not enough studies reported this information. There is some evidence to suspect these factors to influence the association between self-regulation and academic performance (Blair and Raver, 2012; Cadima et al., 2015; Lawson et al., 2018).

An unexpected finding of this meta-analysis is that the HTKS has been utilized mostly with typically developing samples of children. The few studies that did target clinical samples of children included those with learning, language, or behavioral challenges (e.g., Graziano et al., 2016). Despite the significance of this topic, combining clinical samples with samples of children that are predominantly typically developing could produce too much unexplainable heterogeneity in the findings (Wilson, 2019); therefore, we excluded all clinical samples from this meta-analysis. A future approach could be to conduct a meta-analysis examining the self-regulation and academic performance association in studies focused exclusively on children with learning or behavioral challenges.

An additional limitation of this study is that it was not possible to establish causation. In other words, because most of the research is cross-sectional and non-experimental, we cannot confirm that the HTKS is truly predictive of children's academic achievement. For instance, the HTKS and academic performance association may exist because a third variable, such as processing speed or access to developmentally enriching experiences, is either enhancing or compromising children's behavioral regulation and academic skills concomitantly. Therefore, more research is needed to fully understand the validity and utility of the HTKS in practice settings. On a similar note, it is important to consider how the coding of the variables could have played a role in the results. For example, like most meta-analyses, the current study's age variable was represented by the mean age of the sample. One short-coming of this approach is that it doesn't take into account age range variation of the selected studies. Future research should examine whether age range variation both within and between studies influences the association between the HTKS and children's academic performance. Several of the studies took place in preschool settings where children's ages ranged from ~36 to 60 months. Considering the rapid development of children's self-regulation skills during the preschool years, this relatively wide range could have inflated the HTKS and academic association in those particular studies.

Finally, another limitation of this study is that we did not include the gray literature, a decision influenced much by constraints related to time and resources. This practice could increase the risk of publication bias in the findings of this meta-analysis. However, there are advantages associated with excluding gray literature that should also be considered. First, searching and retrieving unpublished studies is more resource intensive and involves methods that are often less reliable and reproducible than traditional database searching (Adams et al., 2016; Hartling et al., 2017). In addition, though encouraged by some meta-analysis guidelines and standards, there is limited empirical support that including unpublished studies and dissertations in a meta-analysis alters the results significantly (Hartling et al., 2017).

We conclude that despite the limitations above, this study has several strengths. The work represents the first meta-analysis of the predictive properties of the HTKS. Thus, it extends knowledge about the measurement characteristics of an instrument that researchers rely on frequently to make claims about children's self-regulation. The findings also provide further evidence that when self-regulation is assessed with a valid and reliable measure, children's self-regulation skills contribute significantly and consistently to educational outcomes. Finally, a notable strength of this work lies in the transparency of the methods. Future researchers could, therefore, easily extend or replicate the research to address some of the shortcomings mentioned.

## Data availability statement

The original contributions presented in the study are included in the article/Supplementary material. Further inquiries can be directed to the corresponding author.

## Author contributions

SK performed the statistical analysis, developed the codebooks, and wrote the first draft of the manuscript. SK and CC contributed to the conception of the study and wrote the second draft. SK, CC, and MM contributed to the design. SK and JK managed/organized the data and conducted the quality appraisal of the studies. AA contributed to the presentation of results. AA, JK, and PB helped with the abstract screen/full-text review and data extraction/coding. MM contributed to the literature review of the second draft. All authors contributed to manuscript revision, read, and approved the submitted version.
