# Supplementary figures and images for: A meta-analysis of the validity of the Head-Toes-Knees-Shoulders task in predicting young children's academic performance
Source: Front Psychol. 2023 Jun 20;14:1124235. doi: 10.3389/fpsyg.2023.1124235 (PMC10319628; doi:10.3389/fpsyg.2023.1124235)

Supplementary Material

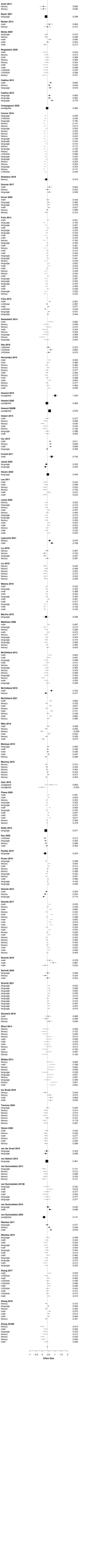

Supplement: Supplementary file 1 [file Data_Sheet_1.PDF]
