# Supplementary material for: A meta-analysis of the validity of the Head-Toes-Knees-Shoulders task in predicting young children's academic performance
Source: Front Psychol. 2023 Jun 20;14:1124235. doi: 10.3389/fpsyg.2023.1124235 (PMC10319628; doi:10.3389/fpsyg.2023.1124235)
Supplement: Supplementary file 2 [file Data_Sheet_2.docx]

# **Appendix A: Publications Included in the Quantitative Analysis**

Allan, D. M., Allan, N. P., Lerner, M. D., Farrington, A. L., & Lonigan, C. J. (2015). Identifying unique components of preschool children's self-regulatory skills using executive function tasks and continuous performance tests. *Early childhood research quarterly, 32*, 40-50. <https://doi.org/10.1016/j.ecresq.2015.02.001>

Ansari, A., Pianta, R. C., Whittaker, J. V., Vitiello, V. E., & Ruzek, E. A. (2020). Persistence and convergence: The end of kindergarten outcomes of pre-K graduates and their nonattending peers. *Developmental Psychology, 56*(11), 2027–2039. <https://doi.org/10.1037/dev0001115>

Aram, D., Abiri, S., & Elad, L. (2014). Predicting early spelling: The contribution of children’s early literacy, private speech during spelling, behavioral regulation, and parental spelling support. *Reading and Writing, 27*(4), 685–707. <https://doi.org/10.1007/s11145-013-9466-z>

Bauer, R. H., Gilpin, A. T., & Thibodeau-Nielsen, R. B. (2021). Executive functions and imaginative play: Exploring relations with prosocial behaviors using structural equation modeling. *Trends in Neuroscience and Education, 25,* 100165. <https://doi.org/10.1016/j.tine.2021.100165>

Becker, D. R., McClelland, M. M., Loprinzi, P., & Trost, S. G. (2014). Physical Activity, Self-Regulation, and Early Academic Achievement in Preschool Children. *Early Education and Development, 25*(1), 56–70. <https://doi.org/10.1080/10409289.2013.780505>

Beisly, A., Kwon, K. A., & Jeon, S. (2020). Executive function and learning behaviors: associations with academic skills among preschoolers. *Early Child Development and Care, 190*(15), 2469-2483. <https://doi.org/10.1080/03004430.2019.1585347>

Birgisdottir, F., Gestsdottir, S., & Geldhof, G. J. (2020). Early predictors of first and fourth grade reading and math: The role of self-regulation and early literacy skills. *Early Childhood Research Quarterly*, 53, 507-519. <https://doi.org/10.1016/j.ecresq.2020.05.001>

Cadima, J., Barros, S., Ferreira, T., Serra-Lemos, M., Leal, T., & Verschueren, K. (2019). Bidirectional associations between vocabulary and self-regulation in preschool and their interplay with teacher–child closeness and autonomy support. *Early Childhood Research Quarterly, 46*, 75–86. <https://doi/10.1016/j.ecresq.2018.04.004>

Cadima, J., Gamelas, A. M., McClelland, M., & Peixoto, C. (2015). Associations between early family risk, children’s behavioral regulation, and academic achievement in Portugal. *Early Education and Development, 26*(5-6), 708–728. <https://doi/10.1080/10409289.2015.1005729>

Compagnoni, M., Sieber, V., & Job, V. (2020). My brain needs a break: kindergarteners’ willpower theories are related to behavioral self-regulation. *Frontiers in psychology, 3567*. <https://doi.org/10.3389/fpsyg.2020.601724>

Connor, C. M., Day, S. L., Phillips, B., Sparapani, N., Ingebrand, S. W., McLean, L., ... & Kaschak, M. P. (2016). Reciprocal effects of self‐regulation, semantic knowledge, and reading comprehension in early elementary school. *Child development, 87*(6), 1813-1824. <https://doi.org/10.1111/cdev.12570>

Distefano, R., Galinsky, E., McClelland, M. M., Zelazo, P. D., & Carlson, S. M. (2018). Autonomy-supportive parenting and associations with child and parent executive function. *Journal of Applied Developmental Psychology*, 58, 77-85. <https://doi.org/10.1016/j.appdev.2018.04.007>

Duncan, R. J., McClelland, M. M., & Acock, A. C. (2017). Relations between executive function, behavioral regulation, and achievement: Moderation by family income. *Journal of Applied Developmental Psychology*, 49, 21–30. <https://doi.org/10.1016/j.appdev.2017.01.004>

Duran, C. A. K., & Grissmer, D. W. (2020). Choosing immediate over delayed gratification correlates with better school-related outcomes in a sample of children of color from low-income families. *Developmental Psychology, 56*(6), 1107–1120. <https://doi.org/10.1037/dev0000920>

Fuhs, M. W., Farran, D. C., & Nesbitt, K. T. (2015). Prekindergarten children’s executive functioning skills and achievement gains: The utility of direct assessments and teacher ratings. *Journal of Educational Psychology, 107*(1), 207–221. <https://doi.org/10.1037/a0037366>

Fuhs, M. W., Nesbitt, K. T., Farran, D. C., & Dong, N. (2014). Longitudinal associations between executive functioning and academic skills across content areas. *Developmental Psychology, 50*(6), 1698–1709. <http://dx.doi.org/10.1037/a0036633>

Gestsdottir, S., von Suchodoletz, A., Wanless, S. B., Hubert, B., Guimard, P., Birgisdottir, F., Gunzenhauser, C., & McClelland, M. (2014). Early Behavioral Self-Regulation, Academic Achievement, and Gender: Longitudinal Findings From France, Germany, and Iceland. *Applied Developmental Science, 18*(2), 90–109. <https://doi.org/10.1080/10888691.2014.894870>

Hee, P. J., Xu, Y., & Krieg, A. (2018). Validation of the Head–Toes–Knees–Shoulders task in Native Hawaiian and non-Hawaiian children. *Early Childhood Research Quarterly, 44*, 192–205. <https://doi.org/10.1016/j.ecresq.2017.12.007>

Hernández, M. M., Eisenberg, N., Valiente, C., Spinrad, T. L., Johns, S. K., Berger, R. H., Silva, K. M., Diaz, A., Gal-Szabo, D. E., Thompson, M. S., & Southworth, J. (2018). Self-Regulation and Academic Measures Across the Early Elementary School Grades: Examining Longitudinal and Bidirectional Associations. *Early Education and Development, 29*(7), 914–938. <https://doi.org/10.1080/10409289.2018.1496722>

Howard, S. J., & Vasseleu, E. (2020). Self-Regulation and Executive Function Longitudinally Predict Advanced Learning in Preschool. *Frontiers in Psychology, 11*, 49. <https://doi.org/10.3389/fpsyg.2020.00049>

Howard, S. J., Neilsen-Hewett, C., de Rosnay, M., Vasseleu, E., & Melhuish, E. (2019). Evaluating the viability of a structured observational approach to assessing early self-regulation. *Early Childhood Research Quarterly, 48,* 186–197. <https://doi.org/10.1016/j.ecresq.2019.03.003>

Howard, S. J., Vasseleu, E., Batterham, M., & Neilsen-Hewett, C. (2020). Everyday practices and activities to improve pre-school self-regulation: cluster RCT evaluation of the PRSIST program. *Frontiers in Psychology, 11*, 137. <https://doi.org/10.3389/fpsyg.2020.00137>

Hubert, B., Guimard, P., Florin, A., & Tracy, A. (2015). Indirect and Direct Relationships Between Self-Regulation and Academic Achievement During the Nursery/Elementary School Transition of French Students. *Early Education and Development, 26(5-6),* 685–707. <https://doi.org/10.1080/10409289.2015.1037624>

Hur, E., Buettner, C. K., & Jeon, L. (2015). The Association Between Teachers’ Child-Centered Beliefs and Children’s Academic Achievement: The Indirect Effect of Children’s Behavioral Self-regulation. *In Child & Youth Care Forum* (Vol. 44, Issue 2, pp. 309–325). <https://doi.org/10.1007/s10566-014-9283-9>

Ivrendi, A. (2011). Influence of self-regulation on the development of children’s number sense. *Early Childhood Education Journal, 39(*4), 239–247. <https://doi.org/10.1007/s10643-011-0462-0>

Jacob, L., Benick, M., Dörrenbächer, S., & Perels, F. (2020). Promoting self-regulated learning in preschoolers. *Journal of Childhood, Education & Society, 1*(2), 116-140. <https://doi.org/10.37291/2717638X.20201237>

Keown, L. J., Franke, N., & Triggs, C. M. (2020). An Evaluation of a Classroom-Based Intervention to Improve Executive Functions in 4-Year Old Children in New Zealand. *Early Childhood Education Journal, 48*(5), 621–631. <https://doi.org/10.1007/s10643-020-01023-x>

Lan, X., Legare, C. H., Ponitz, C. C., Li, S., & Morrison, F. J. (2011). Investigating the links between the subcomponents of executive function and academic achievement: a cross-cultural analysis of Chinese and American preschoolers. *Journal of Experimental Child Psychology, 108*(3), 677–692. (US Sample) <https://doi.org/10.1016/j.jecp.2010.11.001>

Lenes, R., McClelland, M. M., ten Braak, D., Idsøe, T., & Størksen, I. (2020). Direct and indirect pathways from children’s early self-regulation to academic achievement in fifth grade in Norway. *Early Childhood Research Quarterly, 53*, 612–624. <https://doi.org/10.1016/j.ecresq.2020.07.005>

Lipscomb, S. T., Hatfield, B., Lewis, H., Goka-Dubose, E., & Abshire, C. (2021). Adverse childhood experiences and children's development in early care and education programs. *Journal of Applied Developmental Psychology, 72*, 101218. <https://doi.org/10.1016/j.appdev.2020.101218>

Liu, C., Chung, K. K. H., & Fung, W. K. (2019). Bidirectional relationships between children’s executive functioning, visual skills, and word reading ability during the transition from kindergarten to primary school. *Contemporary Educational Psychology, 59*, 101779. <https://doi.org/10.1016/j.cedpsych.2019.101779>

Liu, Y., Sun, H., Lin, D., Li, H., Yeung, S. S.‐s., & Wong, T. T.‐Y. (2018). The unique role of executive function skills in predicting Hong Kong kindergarteners’ reading comprehension. *British Journal of Educational Psychology, 88*(4), 628–644. <https://doi.org/10.1111/bjep.12207>

Malone, S. A., Burgoyne, K., & Hulme, C. (2019). Number knowledge and the approximate number system are two critical foundations for early arithmetic development. *Journal of Educational Psychology, 112*(6), 1167–1182. <https://doi.org/10.1037/edu0000426>

Martins, E. C., Osório, A., Veríssimo, M., & Martins, C. (2016). Emotion understanding in preschool children: The role of executive functions. *International Journal of Behavioral Development, 40*(1), 1-10. <https://doi.org/10.1177/0165025414556>

Matthews, J. S., Ponitz, C. C., & Morrison, F. J. (2009). Early gender differences in self-regulation and academic achievement. Journal of Educational Psychology, 101(3), 689–704. <https://doi.org/10.1037/a0014240>

McClelland, M. M., & Wanless, S. B. (2012). Growing Up With Assets and Risks: The Importance of Self-Regulation for Academic Achievement. *Research in Human Development, 9*(4), 278–297. <https://doi.org/10.1080/15427609.2012.729907>

McClelland, M. M., Gonzales, C. R., Cameron, C. E., Geldhof, G. J., Bowles, R. P., Nancarrow, A. F., ... & Tracy, A. (2021). The Head-Toes-Knees-Shoulders revised: Links to academic outcomes and measures of EF in young children. Frontiers in Psychology, 12. <https://doi.org/10.3389/fpsyg.2021.721846>

McClelland, M. M., Tominey, S. L., Schmitt, S. A., Hatfield, B. E., Purpura, D. J., Gonzales, C. R., & Tracy, A. N. (2019). Red Light, Purple Light! Results of an Intervention to Promote School Readiness for Children From Low-Income Backgrounds. *Frontiers in Psychology, 10*, 2365. <https://doi.org/10.3389/fpsyg.2019.02365>

Mills, B., Dyer, N., Pacheco, D., Brinkley, D., Owen, M. T., & Caughy, M. O. (2019). Developmental Transactions Between Self‐Regulation and Academic Achievement Among Low‐Income African American and Latino Children. *Child Development, 90*(5), 1614–1631. <https://doi.org/10.1111/cdev.13091>

Montoya, M. F., Susperreguy, M. I., Dinarte, L., Morrison, F. J., San Martín, E., Rojas-Barahona, C. A., & Förster, C. E. (2019). Executive function in Chilean preschool children: Do short-term memory, working memory, and response inhibition contribute differentially to early academic skills? *Early Childhood Research Quarterly, 46*, 187–200. <https://doi.org/10.1016/j.ecresq.2018.02.009>

Montroy, J. J., Bowles, R. P., & Skibbe, L. E. (2016). The effect of peers’ self-regulation on preschooler's self-regulation and literacy growth. *Journal of Applied Developmental Psychology, 46*, 73–83. <https://doi.org/10.1016/j.appdev.2016.09.001>

Özer, S. (2016). Behavioral regulation, visual spatial maturity in kindergarten, and the relationship of school adaptation in the first grade for a sample of Turkish children. *Psychological Reports, 118*(2), 353–371. <https://doi.org/10.1177/0033294116633356>

Pianta, R. C., Whittaker, J. E., Vitiello, V., Ruzek, E., Ansari, A., Hofkens, T., & DeCoster, J. (2020). Children’s school readiness skills across the pre-K year: Associations with teacher-student interactions, teacher practices, and exposure to academic content. *Journal of Applied Developmental Psychology, 66.* <https://doi.org/10.1016/j.appdev.2019.101084>

Reilly, S. E., & Downer, J. T. (2019). Roles of executive functioning and language in developing low-income preschoolers’ behavior and emotion regulation. *Early childhood research quarterly, 49*, 229-240. https://doi 10.1016/j.ecresq.2019.07.006

Ren, L., Cheung, R. Y., Boise, C., Li, X., & Fan, J. (2020). Fathers’ perceived co-parenting and children's academic readiness among Chinese preschoolers: Longitudinal pathways through parenting and behavioral regulation. *Early Childhood Research Quarterly, 53*, 77-85. <https://doi.org/10.1016/j.ecresq.2020.03.005>

Rowles, S. P., & Mills, C. M. (2019). “Is it worth my time and effort?”: How children selectively gather information from experts when faced with different kinds of costs. *Journal of Experimental Child Psychology, 179*, 308-323. <https://doi.org/10.1016/j.jecp.2018.11.016>

Russo, J. M., Williford, A. P., Markowitz, A. J., Vitiello, V. E., & Bassok, D. (2019). Examining the validity of a widely-used school readiness assessment: Implications for teachers and early childhood programs. *Early Childhood Research Quarterly, 48*, 14–25. <https://doi.org/10.1016/j.ecresq.2019.02.003>

Schmitt, S. A., Duncan, R. J., Budrevich, A., & Korucu, I. (2020). Benefits of behavioral self-regulation in the context of high classroom quality for preschoolers’ mathematics. *Early Education and Development, 31(*3), 323-334. <https://doi.org/10.1080/10409289.2019.1660555>

Schmitt, S. A., Finders, J. K., Duncan, R. J., Korucu, I., Bryant, L. M., Purpura, D. J., & Elicker, J. G. (2021). Examining transactional relations between behavioral self-regulation and social-emotional functioning during the transition to kindergarten. *Developmental Psychology, 57*(12), 2093. <https://doi.org/10.1037/dev0001266>

Schmitt, S. A., Geldhof, G. J., Purpura, D. J., Duncan, R., & McClelland, M. M. (2017). Examining the relations between executive function, math, and literacy during the transition to kindergarten: A multi-analytic approach. *Journal of Educational Psychology, 109*(8), 1120–1140. <https://doi.org/10.1037/edu0000193>

Schmitt, S. A., Korucu, I., Napoli, A. R., Bryant, L. M., & Purpura, D. J. (2018). Using block play to enhance preschool children’s mathematics and executive functioning: A randomized controlled trial. *Early Childhood Research Quarterly, 44*, 181–191. <https://doi.org/10.1016/j.ecresq.2018.04.006>

Schmitt, S. A., McClelland, M. M., Tominey, S. L., & Acock, A. C. (2015). Strengthening school readiness for Head Start children: Evaluation of a self-regulation intervention. *Early Childhood Research Quarterly, 30,* 20–31. <https://doi.org/10.1016/j.ecresq.2014.08.001>

Schwarz, M., & Shaul, S. (2018). Towards a better understanding of the link between executive functions, early literacy, and emergent mathematical abilities. *Written Language and Literacy, 21*(2), 238–268. <https://doi.org/10.1075/wll.00016.sch>

Shaul, S., & Schwartz, M. (2014). The role of the executive functions in school readiness among preschool-age children. *Reading and Writing, 27*(4), 749–768. <https://doi.org/10.1007/s11145-013-9470-3>

Skibbe, L. E., Hindman, A. H., Connor, C. M., Housey, M., & Morrison, F. J. (2013). Relative Contributions of Pre-Kindergarten and Kindergarten to Children’s Literacy and Mathematics Skills. *Early Education and Development, 24*(5), 687–703. <https://doi.org/10.1080/10409289.2012.712888>

ten Braak, D., Kleemans, T., Størksen, I., Verhoeven, L., & Segers, E. (2018). Domain-specific effects of attentional and behavioral control in early literacy and numeracy development. *Learning and Individual Differences, 68*, 61–71. <https://doi.org/10.1016/j.lindif.2018.10.001>

Traverso, L., Viterbori, P., Gandolfi, E., Zanobini, M., & Usai, M. C. (2022). The contribution of inhibitory control to early literacy skills in 4‐to 5‐year‐old children. *Early Childhood Research Quarterly, 59*, 265-286. <https://doi.org/10.1016/j.ecresq.2021.11.010>

Valcan, D. S., Davis, H. L., Pino-Pasternak, D., & Malpique, A. A. (2020). Executive functioning as a predictor of children’s mathematics, reading and writing. *Journal of Applied Developmental Psychology, 70*(101196), 101196. <https://doi.org/10.1016/j.appdev.2020.101196>

van der Graaf, J., Segers, E., & Verhoeven, L. (2018). Individual differences in the development of scientific thinking in kindergarten. *Learning and Instruction, 56,* 1–9. <https://doi.org/10.1016/j.learninstruc.2018.03.005>

von Salisch, M., Haenel, M., & Denham, S. A. (2015). Self-Regulation, Language Skills, and Emotion Knowledge in Young Children From Northern Germany. *Early Education and Development, 26*(5-6), 792–806. <https://doi.org/10.1080/10409289.2015.994465>

Von Suchodoletz, A., & Gunzenhauser, C. (2013B). Behavior regulation and early math and vocabulary knowledge in German preschool children. *Early Education and Development, 24*(3), 310–331. <https://doi.org/10.1080/10409289.2012.693428>

von Suchodoletz, A., Barza, L., & Larsen, R. A. A. (2020). Examination of teacher–child interactions in early childhood education programmes in the United Arab Emirates. *International Journal of Early Years Education, 28*(1), 6–21. <https://doi.org/10.1080/09669760.2019.1594720>

von Suchodoletz, A., Gestsdottir, S., Wantless, S. B., McClelland, M. M., Birgisdottir, F., Gunzenhauser, C., & Ragnarsdottir, H. (2013A). Behavioral self-regulation and relations to emergent academic skills among children in Germany and Iceland. *Early Childhood Research Quarterly, 28*(1), 62–73. (Iceland first grade sample) <https://doi.org/10.1016/j.ecresq.2012.05.003>

Von Suchodoletz, A., Uka, F., & Larsen, R. A. (2015). Self-regulation across different contexts: Findings in young Albanian children. *Early Education and Development, 26*(5-6), 829–846. <https://doi.org/10.1080/10409289.2015.1012189>

Wanless, S. B., Kim, K. H., Zhang, C., Degol, J. L., Chen, J. L., & Chen, F. M. (2016). Trajectories of behavioral regulation for Taiwanese children from 3.5 to 6 years and relations to math and vocabulary outcomes. *Early Childhood Research Quarterly, 34*, 104–114. <https://doi.org/10.1016/j.ecresq.2015.10.001>

Wanless, S. B., McClelland, M. M., Acock, A. C., Ponitz, C. C., Son, S.-H., Lan, X., Morrison, F. J., Chen, J.-L., Chen, F.-M., Lee, K., Sung, M., & Li, S. (2011). Measuring behavioral regulation in four societies. *Psychological Assessment, 23*(2), 364–378 (South Korean sample) <https://doi.org/10.1037/a0021768>

Zhang, L., & Rao, N. (2017). Effortful Control and Academic Achievement in Rural China. *Early Education and Development, 28*(5), 541–558. <https://doi.org/10.1080/10409289.2016.1255080>

Zhang, X. (2018B). name knowledge longitudinally predicts young Chinese children's Chinese word reading and number competencies in a multilingual context. *Learning and Individual Differences, 65*, 176-186. <https://doi.org/10.1016/j.lindif.2018.06.004>

Zhang, X., Hu, B. Y., Ren, L., & Fan, X. (2018A). Sources of individual differences in young Chinese children’s reading and mathematics skill: A longitudinal study. *Journal of School Psychology, 71*, 122–137. <https://doi.org/10.1016/j.jsp.2018.10.008>
