# Supplementary material for: A meta-analysis of the validity of the Head-Toes-Knees-Shoulders task in predicting young children's academic performance
Source: Front Psychol. 2023 Jun 20;14:1124235. doi: 10.3389/fpsyg.2023.1124235 (PMC10319628; doi:10.3389/fpsyg.2023.1124235)
Supplement: Supplementary file 3 [file Data_Sheet_3.docx]

# **Appendix B: Publications Excluded with Reasons**

| **Number** | **References** | **Reasons For Exclusion** |
| --- | --- | --- |
| 1 | Adam, N., Blaye, A., Gulbinaite, R., Chabé-Ferret, S., & Farrer, C. (2022). A multidimensional evaluation of the benefits of an ecologically realistic training based on pretend play for preschoolers’ cognitive control and self-regulation: From behavior to the underlying theta neuro-oscillatory activity. Journal of Experimental Child Psychology, 216, 105348. | Could not obtain 0-order correlations from researchers |
| 2 | Allan, N. P., & Lonigan, C. J. (2011). Examining the dimensionality of effortful control in preschool children and its relation to academic and socioemotional indicators. Developmental psychology, 47(4), 905. | Could not obtain 0-order correlations from researchers |
| 3 | Allan, N. P., & Lonigan, C. J. (2014). Exploring dimensionality of effortful control using hot and cool tasks in a sample of preschool children. Journal of experimental child psychology, 122, 33-47. | Researcher designed and administered an experimental version of the HTKS with rewards/prizes |
| 4 | Ansari, A., Pianta, R. C., Whittaker, J. E., Vitiello, V., & Ruzek, E. (2021). Enrollment in public-prekindergarten and school readiness skills at kindergarten entry: Differential associations by home language, income, and program characteristics. Early Childhood Research Quarterly, 54, 60-71. | 0-order correlations provided in another included article |
| 5 | Ansari, A., Pianta, R. C., Whittaker, J. V., Vitiello, V. E., & Ruzek, E. A. (2019). Starting Early: The Benefits of Attending Early Childhood Education Programs at Age 3. American Educational Research Journal, 56(4), 1495–1523. | 0-order correlations provided in another included article |
| 6 | Arredondo, M. M., Hu, X.-S., Satterfield, T., & Kovelman, I. (2017). Bilingualism alters children’s frontal lobe functioning for attentional control. Developmental Science, 20(3). https://doi.org/10.1111/desc.12377 | Children are older than 8 years old at the time of HTKS administration |
| 7 | Baptista, J., Osório, A., Martins, E. C., Castiajo, P., Barreto, A. L., Mateus, V., ... & Martins, C. (2017). Maternal and paternal mental‐state talk and executive function in preschool children. Social Development, 26(1), 129-145. | 0-order correlations provided in another included article |
| 8 | Baptista, J., Sousa, D., Soares, I., & Martins, C. (2018). Fathers’ sensitive guidance moderates the association between coparenting and behavioral regulation in preschoolers. International Journal of Behavioral Development, 42(6), 574-580. | 0-order correlations provided in another included article |
| 9 | Becker, D. R., Miao, A., Duncan, R., & McClelland, M. M. (2014). Behavioral self-regulation and executive function both predict visuomotor skills and early academic achievement. Early Childhood Research Quarterly, 29(4), 411–424. | 0-order correlations provided in another included article |
| 10 | Berkes, J., Raikes, A., Bouguen, A., & Filmer, D. (2019). Joint roles of parenting and nutritional status for child development: Evidence from rural Cambodia. Developmental Science, 22(5), e12874. | Could not obtain 0-order correlations from researchers |
| 11 | Birgisdóttir, F., Gestsdóttir, S., & Thorsdóttir, F. (2015). The Role of Behavioral Self-Regulation in Learning to Read: A 2-Year Longitudinal Study of Icelandic Preschool Children. Early Education and Development, 26(5-6), 807–828. | 0-order correlations provided in another included article |
| 12 | Brock, L. L., Kim, H., & Grissmer, D. W. (2018). Longitudinal Associations Among Executive Function, Visuomotor Integration, and Achievement in a High-Risk Sample. Mind, Brain and Education: The Official Journal of the International Mind, Brain, and Education Society, 12(1), 23–27. | 0-order correlations provided in another included article |
| 13 | Brock, L. L., Kim, H., Gutshall, C. C., & Grissmer, D. W. (2018). The development of theory of mind: Predictors and moderators of improvement in kindergarten. Early Child Development and Care. | 0-order correlations provided in another included article |
| 14 | Brock, L. L., Kim, H., Kelly, C. L., Mashburn, A. J., & Grissmer, D. W. (2019). Theory of mind, directly and indirectly, facilitates kindergarten adjustment via verbal ability, executive function, and emotion knowledge. Psychology in the Schools, 56(2), 176-193. | 0-order correlations provided in another included article |
| 15 | Burrage, M. S., Ponitz, C. C., McCready, E. A., Shah, P., Sims, B. C., Jewkes, A. M., & Morrison, F. J. (2008). Age- and Schooling-Related Effects on Executive Functions in Young Children: A Natural Experiment. In Child Neuropsychology (Vol. 14, Issue 6, pp. 510–524). | Could not obtain 0-order correlations from researchers |
| 16 | Cadima, J., Verschueren, K., Leal, T., & Guedes, C. (2016). Classroom interactions, dyadic teacher–child relationships, and self–regulation in socially disadvantaged young children. Journal of abnormal child psychology, 44(1), 7-17. | 0-order correlations provided in another included article |
| 17 | Cameron, C. E., & Morrison, F. J. (2011). Teacher activity orienting predicts preschoolers’ academic and self-regulatory skills. Early Education and Development, 22(4), 620–648. | 0-order correlations provided in another included article |
| 18 | Cameron, C. E., Brock, L. L., Murrah, W. M., Bell, L. H., Worzalla, S. L., Grissmer, D., & Morrison, F. J. (2012). Fine motor skills and executive function both contribute to kindergarten achievement. Child Development, 83(4), 1229–1244. | 0-order correlations provided in another included article |
| 19 | Cameron, C. E., Kim, H., Duncan, R. J., Becker, D. R., & McClelland, M. M. (2019). Bidirectional and co-developing associations of cognitive, mathematics, and literacy skills during kindergarten. Journal of Applied Developmental Psychology, 62, 135–144. | 0-order correlations provided in another included article |
| 20 | Chandler, M. C., Gerde, H. K., Bowles, R. P., McRoy, K. Z., Pontifex, M. B., & Bingham, G. E. (2021). Self-regulation moderates the relationship between fine motor skills and writing in early childhood. Early Childhood Research Quarterly, 57, 239-250. | Does not contain a literacy, language, or mathematics outcome |
| 21 | Collins, M. A., & Laski, E. V. (2015). Preschoolers’ strategies for solving visual pattern tasks. Early Childhood Research Quarterly, 32, 204–214. | Does not contain a literacy, language, or mathematics outcome |
| 22 | Compagnoni, M., Karlen, Y., & Maag Merki, K. (2019). Play it safe or play to learn: mindsets and behavioral self-regulation in kindergarten. Metacognition and Learning, 14(3), 291-314. | Researchers calculated Spearman correlations, not 0-order correlations |
| 23 | Connor, C. M., Ponitz, C. C., Phillips, B. M., Travis, Q. M., Glasney, S., & Morrison, F. J. (2010). First graders’ literacy and self-regulation gains: The effect of individualizing student instruction. Journal of School Psychology, 48(5), 433–455. | 0-order correlations provided in another included article |
| 24 | Courtier, P., Gardes, M. L., Van der Henst, J. B., Noveck, I. A., Croset, M. C., Epinat‐Duclos, J., ... & Prado, J. (2021). Effects of Montessori Education on the Academic, Cognitive, and Social Development of Disadvantaged Preschoolers: A Randomized Controlled Study in the French Public‐School System. Child development, 92(5), 2069-2088. | Could not obtain 0-order correlations from researchers |
| 25 | Cozzani, F., Usai, M. C., & Zanobini, M. (2013). Linguistic abilities and executive function in the third year of life. Rivista Di Psicolinguistica Applicata/journal of Applied Psycholinguistics, 13(1), 25–43. | Children younger than 3 years old at time of HTKS testing |
| 26 | Daniels, D. H. (2014). Children's affective orientations in preschool and their initial adjustment to kindergarten. Psychology in the Schools, 51(3), 256-272. | Does not contain a literacy, language, or mathematics outcome |
| 27 | Day, S. L., Connor, C. M., & McClelland, M. M. (2015). Children’s behavioral regulation and literacy: The impact of the first grade classroom environment. Journal of School Psychology, 53(5), 409–428. | 0-order correlations provided in another included article |
| 28 | Dombek, J. L., & Connor, C. M. (2012). Preventing retention: First grade classroom instruction and student characteristics. Psychology in the Schools, 49(6), 568–588. | 0-order correlations provided in another included article |
| 29 | Duncan, R. J., Schmitt, S. A., Burke, M., & McClelland, M. M. (2018). Combining a kindergarten readiness summer program with a self-regulation intervention improves school readiness. Early Childhood Research Quarterly, 42, 291–300. | 0-order correlations provided in another included article |
| 30 | Early, D. M., Sideris, J., Neitzel, J., LaForett, D. R., & Nehler, C. G. (2018). Factor structure and validity of the Early Childhood Environment Rating Scale—Third Edition (ECERS-3). Early Childhood Research Quarterly, 44, 242–256. | Could not obtain 0-order correlations from researchers |
| 31 | Foy, J. G., & Mann, V. A. (2014). Adaptive cognitive training enhances executive control and visuospatial and verbal working memory in beginning readers. International Education Research, 2(2), 19–43. | Could not obtain 0-order correlations from researchers |
| 32 | Fung, W. K., & Chung, K. K. H. (2019). The roles of social mastery motivation and parental response in preschoolers’ vocabulary knowledge and self-regulation. Early Child Development and Care. | Could not obtain 0-order correlations from researchers |
| 33 | Fung, W. K., Chung, K. K. H., & Lam, C. B. (2020). Mathematics, executive functioning, and visual–spatial skills in Chinese kindergarten children: Examining the bidirectionality. Journal of Experimental Child Psychology, 199, 104923. | Could not obtain 0-order correlations from researchers |
| 34 | Fung, W.-K., & Chung, K. K. H. (2020). The role of socioeconomic status in Chinese word reading and writing among Chinese kindergarten children. Reading and Writing, 33(2), 377–397. | Could not obtain 0-order correlations from researchers |
| 35 | Fung, W.-K., Chung, K. K.-H., & Cheng, R. W.-Y. (2019). Gender Differences in Social Mastery Motivation and Its Relationships to Vocabulary Knowledge, Behavioral Self-Regulation, and Socioemotional Skills. Early Education and Development, 30(2), 280–293. | Could not obtain 0-order correlations from researchers |
| 36 | Gooch, D., Hulme, C., Nash, H. M., & Snowling, M. J. (2014). Comorbidities in preschool children at family risk of dyslexia. Journal of Child Psychology and Psychiatry, and Allied Disciplines, 55(3), 237–246. | Clinical sample |
| 37 | Gooch, D., Thompson, P., Nash, H. M., Snowling, M. J., & Hulme, C. (2016). The development of executive function and language skills in the early school years. Journal of Child Psychology and Psychiatry, and Allied Disciplines, 57(2), 180–187. | Clinical sample |
| 38 | Graziano, P. A., & Hart, K. (2016). Beyond behavior modification: Benefits of social–emotional/self-regulation training for preschoolers with behavior problems. Journal of school psychology, 58, 91-111. | Clinical sample |
| 39 | Graziano, P. A., Garb, L. R., Ros, R., Hart, K., & Garcia, A. (2016). Executive Functioning and School Readiness Among Preschoolers With Externalizing Problems: The Moderating Role of the Student–Teacher Relationship. Early Education and Development, 27(5), 573–589. | Clinical sample |
| 40 | Graziano, P. A., Slavec, J., Hart, K., Garcia, A., & Pelham, W. E. (2014). Improving School Readiness in Preschoolers with Behavior Problems: Results from a Summer Treatment Program. Journal of Psychopathology and Behavioral Assessment, 36(4), 555–569. | Clinical sample |
| 41 | Graziano, P. A., Slavec, J., Ros, R., Garb, L., Hart, K., & Garcia, A. (2015). Self-regulation assessment among preschoolers with externalizing behavior problems. Psychological Assessment, 27(4), 1337–1348. | Clinical sample |
| 42 | Gregg, D. E., Hart, K. C., Vaquerano, S., Cuervo, S., Suarez, M., & Graziano, P. A. (2021). Multidisciplinary Early Intervention for Preschoolers with Externalizing Behavior Problems and Language Impairment: Results from an Open Trial. Journal of Psychopathology and Behavioral Assessment, 43(3), 506-517. | Clinical sample |
| 43 | Gunzenhauser, C., & von Suchodoletz, A. (2014). Preschoolers' use of suppression influences subsequent self-control but does not interfere with verbal memory. Learning and Individual Differences, 32, 219-224. | Could not obtain 0-order correlations from researchers |
| 44 | Hare, M. M., Garcia, A. M., Hart, K. C., & Graziano, P. A. (2021). Intervention response among preschoolers with ADHD: The role of emotion understanding. Journal of School Psychology, 84, 19-31. | Clinical sample |
| 45 | Hart, K. C., Maharaj, A. V., & Graziano, P. A. (2019). Does dose of early intervention matter for preschoolers with externalizing behavior problems? A pilot randomized trial comparing intensive summer programming to school consultation. Journal of School Psychology, 72, 112–133. | clinical sample |
| 46 | Hernández, M. M., Valiente, C., Eisenberg, N., Spinrad, T. L., Berger, R. H., Johns, S. K., ... & Pina, A. A. (2021). Do peer and child temperament jointly predict student–teacher conflict and closeness?. Journal of Applied Developmental Psychology, 76, 101319. | Does not contain a literacy, language, or mathematics outcome |
| 47 | Herold, K. H., Bock, A. M., Murphy, M. M., & Mazzocco, M. M. M. (2019). Expanding task instructions may increase fractions problem difficulty for students with mathematics learning disability. Learning Disability Quarterly: Journal of the Division for Children with Learning Disabilities, 073194871986547. | Children older than 8 years old at time of HTKS testing |
| 48 | Houwen, S., Kamphorst, E., van der Veer, G., & Cantell, M. (2019). Identifying patterns of motor performance, executive functioning, and verbal ability in preschool children: A latent profile analysis. Research in Developmental Disabilities, 84, 3–15. | Researchers calculated Spearman correlations, not 0-order correlations |
| 49 | Hu, B. Y., Fan, X., Wu, Y., LoCasale-Crouch, J., & Song, Z. (2020). Teacher--child interaction quality and Chinese children’s academic and cognitive development: New perspectives from piecewise growth modeling. Early Childhood Research Quarterly, 51, 242–255. | Could not obtain 0-order correlations from researchers |
| 50 | Hu, B. Y., Johnson, G. K., & Wu, H. (2018). Screen time relationship of Chinese parents and their children. Children and Youth Services Review, 94, 659–669. | Could not obtain 0-order correlations from researchers |
| 51 | Hu, B. Y., Wu, H., Winsler, A., Fan, X., & Song, Z. (2020). Parent migration and rural preschool children’s early academic and social skill trajectories in China: Are “left-behind”children really left behind? Early Childhood Research Quarterly, 51, 317–328. | Could not obtain 0-order correlations from researchers |
| 52 | Hubert, B., Guimard, P., & Florin, A. (2017). Cognitive self-regulation and social functioning among French children: A longitudinal study from kindergarten to first grade: Self-regulation and social functioning. PsyCh Journal, 6(1), 57–75. | 0-order correlations provided in another included article |
| 53 | Hur, E., Buettner, C. K., & Jeon, L. (2015). Parental depressive symptoms and children’s school-readiness: The indirect effect of household chaos. Journal of Child and Family Studies, 24(11), 3462–3473. | 0-order correlations provided in another included article |
| 54 | Köckeritz, M., Klinkhammer, J., & von Salisch, M. (2010). The development of emotional understanding and behavioral self-regulation in kindergarten children from German and immigrant families. Praxis der Kinderpsychologie und Kinderpsychiatrie, 59(7), 529-544. | Full text not available in English |
| 55 | Landis, T. D., Hart, K. C., & Graziano, P. A. (2019). Targeting self-regulation and academic functioning among preschoolers with behavior problems: Are there incremental benefits to including cognitive training as part of a classroom curriculum?. Child Neuropsychology, 25(5), 688-704. | 0-order correlations provided in another included article |
| 56 | Lee, M. K., Baker, S., & Whitebread, D. (2018). Culture-specific links between maternal executive function, parenting, and preschool children’s executive function in South Korea. The British Journal of Educational Psychology, 88(2), 216–235. | Researchers calculated Spearman correlations, not 0-order correlations |
| 57 | Lillard, A. S. (2012). Preschool children's development in classic Montessori, supplemented Montessori, and conventional programs. Journal of school psychology, 50(3), 379-401. | Could not obtain 0-order correlations from researchers |
| 58 | Lillard, A. S., Heise, M. J., Richey, E. M., Tong, X., Hart, A., & Bray, P. M. (2017). Montessori preschool elevates and equalizes child outcomes: A longitudinal study. Frontiers in psychology, 1783. | Could not obtain 0-order correlations from researchers |
| 59 | Lipsey, M. W., Nesbitt, K. T., Farran, D. C., Dong, N., Fuhs, M. W., & Wilson, S. J. (2017). Learning-related cognitive self-regulation measures for prekindergarten children: A comparative evaluation of the educational relevance of selected measures. Journal of Educational Psychology, 109(8), 1084–1102. | 0-order correlations provided in another included article |
| 60 | Liu, Y., & Zhang, X. (2022). Spatial skills and counting sequence knowledge: Investigating reciprocal longitudinal relations in early years. Early Childhood Research Quarterly, 59, 1-11. | 0-order correlations provided in another included article |
| 61 | Lonigan, C. J., & Phillips, B. M. (2012). Comparing skills-focused and self-regulation focused preschool curricula: Impacts on academic and self-regulatory skills. Society for Research on Educational Effectiveness. http://files.eric.ed.gov/fulltext/ED530178.pdf | Could not obtain 0-order correlations from researchers |
| 62 | Lonigan, C. J., Allan, D. M., & Phillips, B. M. (2017). Examining the predictive relations between two aspects of self-regulation and growth in preschool children’s early literacy skills. Developmental Psychology, 53(1), 63–76. | Could not obtain 0-order correlations from researchers |
| 63 | Lonigan, C. J., Allan, D. M., Goodrich, J. M., Farrington, A. L., & Phillips, B. M. (2017). Inhibitory control of Spanish-speaking language-minority preschool children: Measurement and association with language, literacy, and math skills. Journal of learning disabilities, 50(4), 373-385. | Could not obtain 0-order correlations from researchers |
| 64 | Lonigan, C. J., Lerner, M. D., Goodrich, J. M., Farrington, A. L., & Allan, D. M. (2016). Executive function of Spanish-speaking language-minority preschoolers: Structure and relations with early literacy skills and behavioral outcomes. Journal of Experimental Child Psychology, 144, 46–65. | Could not obtain 0-order correlations from researchers |
| 65 | Lonigan, Christopher J., Jamie A. Spiegel, J. Marc Goodrich, Brittany M. Morris, Colleen M. Osborne, Matthew D. Lerner, and Beth M. Phillips. "Does preschool self-regulation predict later behavior problems in general or specific problem behaviors?." Journal of abnormal child psychology 45, no. 8 (2017): 1491-1502. | Clinical sample |
| 66 | Lukowski, S. L., Padrutt, E. R., Sarafoglou, K., Ross, J. L., Law, J. R., Olson, R. E., & Mazzocco, M. M. (2020). Variation in early number skills and mathematics achievement: Implications from cognitive profiles of children with or without Turner syndrome. PloS one, 15(10), e0239224. | Clinical sample |
| 67 | Malone, S. A., Pritchard, V. E., Heron-Delaney, M., Burgoyne, K., Lervåg, A., & Hulme, C. (2019). Data on numerosity discrimination, inhibition and arithmetic during the early school years. Data in brief, 25, 104062. | Research brief, data provided in full article |
| 68 | Marcovitch, S., O’Brien, M., Calkins, S. D., Leerkes, E. M., Weaver, J. M., & Levine, D. W. (2015). A longitudinal assessment of the relation between executive function and theory of mind at 3, 4, and 5 years. Cognitive development, 33, 40-55. | Could not obtain 0-order correlations from researchers |
| 69 | Marti, M., Melvin, S., Noble, K. G., & Duch, H. (2018). Intervention fidelity of getting ready for school: associations with classroom and teacher characteristics and preschooler’s school readiness skills. Early Childhood Research Quarterly, 44, 55-71. | Could not obtain 0-order correlations from researchers |
| 70 | McClelland, M. M., Cameron, C. E., Connor, C. M., Farris, C. L., Jewkes, A. M., & Morrison, F. J. (2007). Links between behavioral regulation and preschoolers' literacy, vocabulary, and math skills. Developmental psychology, 43(4), 947. | 0-order correlations provided in another included article |
| 71 | McClelland, M. M., Cameron, C. E., Duncan, R., Bowles, R. P., Acock, A. C., Miao, A., & Pratt, M. E. (2014). Predictors of early growth in academic achievement: The head-toes-knees-shoulders task. Frontiers in psychology, 5, 599. | 0-order correlations provided in another included article |
| 72 | Vitiello, V. E., Bassok, D., Hamre, B. K., Player, D., & Williford, A. P. (2018). Measuring the quality of teacher–child interactions at scale: Comparing research-based and state observation approaches. Early Childhood Research Quarterly, 44, 161-169. | 0-order correlations provided in another included article |
| 73 | Milburn, T. F., Lonigan, C. J., & Phillips, B. M. (2019). Stability of Risk Status During Preschool. Journal of learning disabilities, 52(3), 209-219. | Could not obtain 0-order correlations from researchers |
| 74 | Moll, K., Snowling, M. J., Göbel, S. M., & Hulme, C. (2015). Early language and executive skills predict variations in number and arithmetic skills in children at family-risk of dyslexia and typically developing controls. Learning and Instruction, 38, 53–62. | Clinical sample |
| 75 | Montroy, J. J., Bowles, R. P., Skibbe, L. E., & Foster, T. D. (2014). Social skills and problem behaviors as mediators of the relationship between behavioral self-regulation and academic achievement. Early Childhood Research Quarterly, 29(3), 298–309. | 0-order correlations provided in another included article |
| 76 | Montroy, J. J., Bowles, R. P., Skibbe, L. E., McClelland, M. M., & Morrison, F. J. (2016). The development of self-regulation across early childhood. Developmental psychology, 52(11), 1744. | 0-order correlations provided in another included article |
| 77 | Neal, S. C., Norwalk, K. E., & Haskett, M. E. (2020). Differential impacts of the Incredible Years-Teacher Classroom Management program based on young children’s risk profiles. Early Childhood Research Quarterly, 51, 473-482. | Could not obtain 0-order correlations from researchers |
| 78 | Neha, T., Reese, E., Schaughency, E., & Taumoepeau, M. (2020). The role of whānau (New Zealand Māori families) for Māori children’s early learning. Developmental Psychology, 56(8), 1518. | Could not obtain 0-order correlations from researchers |
| 79 | Nesbitt, K. T., Farran, D. C., & Fuhs, M. W. (2015). Executive function skills and academic achievement gains in prekindergarten: Contributions of learning-related behaviors. Developmental Psychology, 51(7), 865–878. | 0-order correlations provided in another included article |
| 80 | Nesbitt, K. T., Fuhs, M. W., & Farran, D. C. (2019). Stability and instability in the co-development of mathematics, executive function skills, and visual-motor integration from prekindergarten to first grade. Early Childhood Research Quarterly, 46, 262–274. | 0-order correlations provided in another included article |
| 81 | Nguyen, T., Ansari, A., Pianta, R. C., Whittaker, J. V., Vitiello, V. E., & Ruzek, E. (2020). The classroom relational environment and children’s early development in preschool. Social Development, 29(4), 1071-1091. | 0-order correlations provided in another included article |
| 82 | Ntourou, K., Anderson, J. D., & Wagovich, S. A. (2018). Executive function and childhood stuttering: Parent ratings and evidence from a behavioral task. Journal of fluency disorders, 56, 18-32. | Clinical sample |
| 83 | Patricia Pelletier, J., & Corter, J. E. (2019). A longitudinal comparison of learning outcomes in full-day and half-day kindergarten. The Journal of Educational Research, 112(2), 192-210. | Could not obtain 0-order correlations from researchers |
| 84 | Pauli-Pott, U., Schloß, S., Heinzel-Gutenbrunner, M., & Becker, K. (2019). Multiple causal pathways in attention-deficit/hyperactivity disorder–Do emerging executive and motivational deviations precede symptom development?. Child Neuropsychology, 25(2), 179-197. | Clinical sample |
| 85 | Pears, K. C., Healey, C. V., Fisher, P. A., Braun, D., Gill, C., Conte, H. M., Newman, J., & Ticer, S. (2014). Immediate Effects of a Program to Promote School Readiness in Low-Income Children: Results of a Pilot Study. Education & Treatment of Children, 37(3), 431–460. | Could not obtain 0-order correlations from researchers |
| 86 | Pelletier, J., & Fesseha, E. (2019). The impact of full-day kindergarten on learning outcomes and self-regulation among kindergarten children at risk for placement in special education. Exceptionality Education International, 29(3), 42-56. | Could not obtain 0-order correlations from researchers |
| 87 | Ponitz, C. C., McClelland, M. M., Matthews, J. S., & Morrison, F. J. (2009). A structured observation of behavioral self-regulation and its contribution to kindergarten outcomes. Developmental Psychology, 45(3), 605–619. | 0-order correlations provided in another included article |
| 88 | Puranik, C. S., Boss, E., & Wanless, S. (2019). Relations between self-regulation and early writing: Domain specific or task dependent? Early Childhood Research Quarterly, 46, 228–239. | Does not contain a literacy, language, or mathematics outcome |
| 89 | Ren, L., Hu, B. Y., Wu, H., Zhang, X., Davis, A. N., & Hsiao, Y. Y. (2022). Differential associations between extracurricular participation and Chinese children's academic readiness: Preschool teacher–child interactions as a moderator. Early Childhood Research Quarterly, 59, 134-147. | 0-order correlations provided in another included article |
| 90 | Ren, L., Zhang, X., Yang, W., & Song, Z. (2018). Relations among Parenting, Child Behavioral Regulation and Early Competencies: A Study on Chinese Preschoolers. Journal of Child and Family Studies, 27(2), 639–652. | 0-order correlations provided in another included article |
| 91 | Ros, R., & Graziano, P. A. (2020). A transdiagnostic examination of self-regulation: Comparisons across preschoolers with ASD, ADHD, and typically developing children. Journal of Clinical Child & Adolescent Psychology, 49(4), 493-508. | Clinical sample |
| 92 | Ros, R., Graziano, P. A., & Hart, K. C. (2018). Treatment Response among Preschoolers with EBP: The Role of Social Functioning. Journal of psychopathology and behavioral assessment, 40(3), 514-527.tment response among preschoolers with EBP: The role of social functioning. | Clinical sample |
| 93 | Sawyer, C., Adrian, J., Bakeman, R., Fuller, M., & Akshoomoff, N. (2021). Self-regulation task in young school age children born preterm: Correlation with early academic achievement. Early Human Development, 157, 105362. | Clinical sample |
| 94 | Schmitt, S. A., Pratt, M. E., & McClelland, M. M. (2014). Examining the validity of behavioral self-regulation tools in predicting preschoolers’ academic achievement. Early Education and Development, 25(5), 641–660. | 0-order correlations provided in another included article |
| 95 | SEZGİN, E. Y., Leyla, U. L. U. S., & ŞAHİN, İ. The Examination of Relationship Between Behavioral Regulation with Phonological Awareness, Matching Images and Pre-Writing Skills in Preschool Children. Sakarya University Journal of Education, 9(1), 107-128. | Researchers calculated Spearman correlations, not 0-order correlations |
| 96 | Skibbe, L. E., Connor, C. M., Morrison, F. J., & Jewkes, A. M. (2011). Schooling effects on preschoolers’ self-regulation, early literacy, and language growth. Early Childhood Research Quarterly, 26(1), 42–49. | 0-order correlations provided in another article |
| 97 | Skibbe, L. E., Montroy, J. J., Bowles, R. P., & Morrison, F. J. (2019). Self-regulation and the Development of Literacy and Language Achievement from Preschool through Second Grade. Early Childhood Research Quarterly, 46, 240–251. | Could not obtain 0-order correlations from researchers |
| 98 | Skibbe, L. E., Phillips, B. M., Day, S. L., Brophy-Herb, H. E., & Connor, C. M. (2012). Children's early literacy growth in relation to classmates' self-regulation. Journal of educational psychology, 104(3), 541. | 0-order correlations provided in another included article |
| 99 | Snowling, M. J., Gooch, D., McArthur, G., & Hulme, C. (2018). Language skills, but not frequency discrimination, predict reading skills in children at risk of dyslexia. Psychological science, 29(8), 1270-1282. | Clinical sample |
| 100 | Snowling, M. J., Nash, H. M., Gooch, D. C., Hayiou‐Thomas, M. E., Hulme, C., & Wellcome Language and Reading Project Team. (2019). Developmental outcomes for children at high risk of dyslexia and children with developmental language disorder. Child development, 90(5), e548-e564. | Clinical sample |
| 101 | Solomon, T., Plamondon, A., O’Hara, A., Finch, H., Goco, G., Chaban, P., ... & Tannock, R. (2018). A cluster randomized-controlled trial of the impact of the Tools of the Mind curriculum on self-regulation in Canadian preschoolers. Frontiers in psychology, 8, 2366. | Could not obtain 0-order correlations from researchers |
| 102 | Son, S.-H., Lee, K., & Sung, M. (2013). Links between preschoolers’ behavioral regulation and school readiness skills: The role of child gender. Early Education and Development, 24(4), 468–490. | Could not obtain 0-order correlations from researchers |
| 103 | Sparapani, N., Connor, C. M., Day, S., Wood, T., Ingebrand, S., McLean, L., & Phillips, B. (2019). Profiles of foundational learning skills among first graders. Learning and Individual Differences, 70, 216–227. | 0-order correlations provided in another inlcuded article |
| 104 | Spiegel, J. A., & Lonigan, C. J. (2018). A head-to-toes approach to computerized testing of executive functioning in young children. Early Childhood Research Quarterly, 44, 15-23. | Could not obtain 0-order correlations from researchers |
| 105 | Spiegel, J. A., Lonigan, C. J., & Phillips, B. M. (2017). Factor structure and utility of the Behavior Rating Inventory of Executive Function—Preschool Version. Psychological Assessment, 29(2), 172–185. | Could not obtain 0-order correlations from researchers |
| 106 | Tamm, L., & Peugh, J. (2019). Concordance of teacher-rated and performance-based measures of executive functioning in preschoolers. Child Neuropsychology, 25(3), 410-424. | Does not contain a literacy, language, or mathematics outcome |
| 107 | Ten Braak, D., Lenes, R., Purpura, D. J., Schmitt, S. A., & Størksen, I. (2022). Why do early mathematics skills predict later mathematics and reading achievement? The role of executive function. Journal of Experimental Child Psychology, 214, 105306. | 0-order correlations provided in another included article |
| 108 | ten Braak, D., Størksen, I., Idsoe, T., & McClelland, M. (2019). Bidirectionality in self-regulation and academic skills in play-based early childhood education. Journal of Applied Developmental Psychology, 65, 101064. | 0-order correlations presented in another included article |
| 109 | Thompson, P. A., Hulme, C., Nash, H. M., Gooch, D., Hayiou‐Thomas, E., & Snowling, M. J. (2015). Developmental dyslexia: predicting individual risk. Journal of Child Psychology and Psychiatry, 56(9), 976-987. | Clinical sample |
| 110 | Tominey, S. L., & McClelland, M. M. (2011). Red Light, Purple Light: Findings From a Randomized Trial Using Circle Time Games to Improve Behavioral Self-Regulation in Preschool. Early Education and Development, 22(3), 489–519. | Could not obtain 0-order correlations from researchers |
| 111 | Upshur, C. C., Wenz-Gross, M., Rhoads, C., Heyman, M., Yoo, Y., & Sawosik, G. (2019). A randomized efficacy trial of the second step early learning (SSEL) curriculum. Journal of Applied Developmental Psychology, 62, 145-159. | Could not obtain 0-order correlations from researchers |
| 112 | Volckaert, A., & Noël, P. M. P. (2018). Executive function, chaos and temperament: Specificities in preschoolers with externalizing behaviors. Psychologica Belgica, 58(1), 222. | Clinical sample |
| 113 | Voltmer, K., & Von Salisch, M. (2019). Native‐born German and immigrant children's development of emotion knowledge: A latent growth curve analysis. British Journal of Developmental Psychology, 37(1), 112-129. | 0-order correlations provided in another included article |
| 114 | Wang, S., Hu, B. Y., & Zhang, X. (2021). Kindergarteners’ spatial skills and their reading and math achievement in second grade. Early Childhood Research Quarterly, 57, 156-166. | 0-order correlations provided in another included article |
| 115 | Wanless, S. B., McClelland, M. M., Acock, A. C., Chen, F.-M., & Chen, J.-L. (2011). Behavioral Regulation and Early Academic Achievement in Taiwan. Early Education and Development, 22(1), 1–28. | 0-order correlations provided in another included article |
| 116 | Wanless, S. B., McClelland, M. M., Lan, X., Son, S. H., Cameron, C. E., Morrison, F. J., ... & Sung, M. (2013). Gender differences in behavioral regulation in four societies: The United States, Taiwan, South Korea, and China. Early Childhood Research Quarterly, 28(3), 621-633. | Could not obtain 0-order correlations from researchers |
| 117 | Wanless, S. B., McClelland, M. M., Tominey, S. L., & Acock, A. C. (2011). The Influence of Demographic Risk Factors on Children’s Behavioral Regulation in Prekindergarten and Kindergarten. Early Education and Development, 22(3), 461–488. | Could not obtain 0-order correlations from researchers |
| 118 | Wolf, S., & McCoy, D. C. (2019). The role of executive function and social-emotional skills in the development of literacy and numeracy during preschool: a cross-lagged longitudinal study. Developmental Science, 22(4), e12800. | Researchers only administered five HTKS items |
| 119 | Wolf, S., Aber, J. L., Behrman, J. R., & Peele, M. (2019). Longitudinal causal impacts of preschool teacher training on ghanaian children’s school readiness: Evidence for persistence and fade‐out. Developmental science, 22(5), e12878. | Researchers only administered five HTKS items |
| 120 | Xu, C., Burr, S. D. L., Douglas, H., Susperreguy, M. I., & LeFevre, J. A. (2021). Number line development of Chilean children from preschool to the end of kindergarten. Journal of Experimental Child Psychology, 208, 105144. | 0-order correlations provided in another included article |
| 121 | Zhang, C., Bingham, G. E., & Quinn, M. F. (2017). The associations among preschool children’s growth in early reading, executive function, and invented spelling skills. In Reading and Writing (Vol. 30, Issue 8, pp. 1705–1728). https://doi.org/10.1007/s11145-017-9746-0 | Could not obtain 0-order correlations from researchers |
| 122 | Zhang, X. (2016). Linking language, visual-spatial, and executive function skills to number competence in very young Chinese children. Early Childhood Research Quarterly, 36, 178–189. | 0-order correlations provided in another included article |
